# A Method for Producing Highly Pure Magnetosomes in Large Quantity for Medical Applications Using *Magnetospirillum gryphiswaldense* MSR-1 Magnetotactic Bacteria Amplified in Minimal Growth Media

**DOI:** 10.3389/fbioe.2020.00016

**Published:** 2020-02-18

**Authors:** Clément Berny, Raphael Le Fèvre, François Guyot, Karine Blondeau, Christine Guizonne, Emilie Rousseau, Nicolas Bayan, Edouard Alphandéry

**Affiliations:** ^1^Nanobacterie SARL, Paris, France; ^2^Université Paris-Saclay, CEA, CNRS, Institute for Integrative Biology of the Cell (I2BC), Gif-sur-Yvette, France; ^3^Paris Sorbonne Université, Muséum National d'Histoire Naturelle, UMR CNRS 7590, Institut de Minéralogie, de Physique des Matériaux et de Cosmochimie, IMPMC, Paris, France; ^4^Institut Universitaire de France (IUF), Paris, France

**Keywords:** minimal medium, magnetosome, magnetotactic bacteria, metals, yeast extract, iron incorporation

## Abstract

We report the synthesis in large quantity of highly pure magnetosomes for medical applications. For that, magnetosomes are produced by MSR-1 *Magnetospirillum gryphiswaldense* magnetotactic bacteria using minimal growth media devoid of uncharacterized and toxic products prohibited by pharmaceutical regulation, i.e., yeast extract, heavy metals different from iron, and carcinogenic, mutagenic and reprotoxic agents. This method follows two steps, during which bacteria are first pre-amplified without producing magnetosomes and are then fed with an iron source to synthesize magnetosomes, yielding, after 50 h of growth, an equivalent OD_565_ of ~8 and 10 mg of magnetosomes in iron per liter of growth media. Compared with magnetosomes produced in non-minimal growth media, those particles have lower concentrations in metals other than iron. Very significant reduction or disappearance in magnetosome composition of zinc, manganese, barium, and aluminum are observed. This new synthesis method paves the way towards the production of magnetosomes for medical applications.

## Introduction

Iron oxide nanoparticles (IONP) offer a series of appealing properties, such as their faculty to enhance contrast in magnetic resonance imaging (Choi et al., 2004), to efficiently carry drugs (Jain et al., 2005), or to produce localized heat for tumor hyperthermia treatments under the application of an alternating magnetic field or laser (Beik et al., 2016). These properties, together with a reassuring safety profile and good biocompatibility (Arami et al., 2015), have led to their use for various applications in human health, including the treatment or detection of cancers (Jordan et al., 1999). For medical applications, IONP are most commonly composed of a magnetite/maghemite core surrounded by a stabilizing coating, made of various organic or inorganic materials (Teja and Koh, 2009). IONP have been injected in humans in several clinical trials (Maier-Hauff et al., 2011; Renkonen et al., 2017), and are commercialized for the treatment of iron anemia diseases (Auerbach and Macdougall, 2017). Despite a huge progress in the fabrication of these nanoparticles, through various methods such as co-precipitation, electrochemistry, flow injection syntheses, hydrothermal reactions, laser pyrolysis, polyol-iron high temperature reactions, sol-gel methods, sonolysis or thermolysis, spray pyrolysis, as summarized elsewhere (Laurent et al., 2008), it still remains a challenge to develop an IONP production method that meets all the criteria of an ideal synthesis, i.e., a synthesis that: (i) does not use toxic chemicals forbidden by the pharmacopeia, (ii) results in a high production yield, (iii) produces IONP with a sufficiently large size to reach stable ferrimagnetism while avoiding aggregation, (iv) results in a homogenous IONP size distribution, and (v) yields highly pure crystals.

In this study, to reach such ideal conditions, we employed a microbial synthesis method using *Magnetospirillum gryphiswaldense* MSR-1 magnetotactic bacteria (MTB), which are known to be the most productive of MTB strain for bio-mineralizing iron into bio-synthesized IONP called magnetosomes. IONP are used by MTB as a compass to navigate in the direction of the earth magnetic field, using the alignment of IONP magnetic moment in this direction. Using magnetotactism coupled to a system of oxygen detection (aerotactism), MTB then use their flagella to propel themselves toward the oxic-anoxic transition zone, where the oxygen concentration is optimal for their development and for magnetosome synthesis (Lefèvre and Bazylinski, 2013). Magnetosomes consist of magnetite minerals surrounded by a biological membrane (e.g., Faivre and Schüler, 2008; Le Fèvre et al., 2017; Arakaki et al., 2018). Using these bacteria, it has been possible to produce nanoparticles that fulfill most of the criteria mentioned above (Alphandéry, 2014). In particular, MSR-1 magnetites display an organization in chains that prevents their aggregation (Heyen and Schüler, 2003), have high crystallinity, homogenous size distribution and improved heating properties in comparison to chemically synthesized IONP (Hamdous et al., 2017; Mandawala et al., 2017). Hence, these biological nanoparticles have been considered for various medical applications, especially for tumor nanoparticle-mediated hyperthermia treatments (Alphandéry et al., 2017; Le Fèvre et al., 2017). Compared with other species, MSR-1 is the species that can reach the largest magnetosome production yield (MPY), making it the best candidate for producing magnetosomes for medical applications, as reported elsewhere by comparing the MPY obtained from the different MTB species, i.e., MSR-1, AMB-1, MS-1, MV-1 (Ali et al., 2017). However, a few hurdles still need to be overcome to foresee their use in the medical field. First, the large-scale production of magnetosomes is made difficult by the very specific conditions of oxygenation required for growing the MSR-1 strain, which should both be below a first threshold to enable magnetosome synthesis and above a second one to result in efficient bacterial growth. Using a fed-batch growth method, in which iron is brought to the growth medium through an acidic source that maintains the pH at 6.9, a record in magnetosome production yield has been obtained, allowing the reaching of an optical density at 565 nm (OD_565_) of 42 and 356 mg of extracted magnetosomes (in dry weight) per liter of culture medium after 44 h of fermentation (Zhang et al., 2011). This amount of magnetosomes is larger than that of ~40–60 mg/L obtained with other growth protocols known for achieving good yield (Fernández-Castané et al., 2018). Since typical quantities of nanoparticles necessary for a therapeutic treatment are of 1 g or less in iron per patient, as reported for treatments of glioblastoma by magnetic hyperthermia (Maier-Hauff et al., 2011) or iron anemia disease (Auerbach and Macdougall, 2017), about 3 L of bacteria prepared following this method (Zhang et al., 2011) would be sufficient to treat one patient, making this production yield compatible with the use of magnetosomes for medical applications. Despite its high yield, such previously developed growth method suffers from the presence in the bacterial growth medium of some components that are inadvisable in pharmaceuticals designed for human uses. These components are toxic products such as carcinogenic, mutagenic and reprotoxic (CMR) chemicals used as chelating agents (boric acid and nitrilotriacetic acid trisodium salt), heavy metals, and also uncharacterized products coming from animal derived ingredients (Yeast extract) (ICH, 2019).

Moreover, according to pharmaceutical guideline ICH Q3D (ICH, 2019), it is better if pharmaceuticals for human use don't contain metals (and/or metalloid) considered to be toxic beyond an individually defined concentration. We have therefore measured the relative abundance of the metals Ag, Al, As, Ba, Cd, Co, Cr, Cu, Mn, Mo, Ni, Pb, Sb, Se, Si, Ti, Tl, W, and Zn, in extracted magnetosomes at different time points of the growth and adjusted growth media to decrease the amounts of these metals in the final products.

In order to obtain large quantities of “*highly pure magnetosomes,”* which are defined as magnetosomes with the lowest possible quantity of “*impurities”* (other metals than iron) according to pharmaceutical standards, we decided to develop a minimal growth medium enabling bacterial growth and production of chains of magnetosomes, with less components as compared with that of Zhang et al. (2011).

Our methodology to determine this minimal growth medium from a pre-existing medium was inspired by Premaratne et al. (1993). The difficulty lies in being able to simplify the growth media without weakening the growth of magnetotactic bacteria and without inhibiting magnetosome production. This task was not won in advance. Indeed, some of the metals that we needed to eliminate like cobalt have been reported to be necessary for biosynthesis of the B12 vitamin and in some key enzymes responsible for magnetosome formation in AMB-1 strain (Li et al., 2016). Furthermore, according to previous reports, other metals like copper, manganese and zinc, could be essential since they are agents acting as cofactors of bacterial antioxidant defense enzymes (Agranoff and Sanjeev, 1998), thus preventing oxidative stress in bacteria and modifying magnetosomes characteristics (Popa and Fang, 2009). Similarly, removing yeast extract (YE) which contains vitamins known as antioxidants involved in numerous protein biosynthesis pathway, could reduce activity of proteins such as MamGFDC, which are essential in magnetosome size regulation (Scheffel et al., 2008).

In a second part of this work, we present a detailed follow-up of the incorporation of elementary atoms in magnetosomes at different steps of bacterial growth. This study thus shows the production of magnetosomes using a minimal growth medium, which is depleted in non-strictly essential products, while keeping a high production yield and similar magnetosome properties as those obtained in the best reported growth conditions (Zhang et al., 2011). Altogether, our data provide for the first time a synthesis method that achieves highly pure magnetosomes with a very low level of impurities.

## Results

### Development of Minimal Growth Media

For large-scale medical applications, magnetosomes should be produced at the highest possible yield within the shortest period of time. In this respect, the best reported growth conditions are described by Zhang et al. (2011). However, to use the Zhang et al. method for producing magnetosomes for medical applications, the growth conditions need to be adapted by removing toxic and uncharacterized products from the three different growth media, i.e., the pre-growth medium used to amplify magnetotactic bacteria without magnetosome chains production, the growth medium that serves to amplify magnetotactic bacteria and to produce magnetosomes during the growth step, and the fed-batch medium that supplements the growth medium to maintain the pH of the growth medium at 6.9 while bringing an iron source to the growth medium that triggers magnetosome production.

Therefore, we first optimized the pre-growth and growth media by growing MSR-1 magnetotactic bacteria using a two steps cultivation method, mimicking the conditions of the pre-growth and growth steps, respectively, as described in “*Materials and Methods.”* We removed from the three media all constituents that are not compatible with medical regulation (YE, heavy metals apart from iron, and CMR agents) and we determined the lowest concentration of remaining chemicals that enables bacterial growth and magnetosome production to yield “*highly pure magnetosomes.”* To assess bacterial growth and magnetosome production, we grew bacteria in 50 mL tubes (See *“Material and Method”*) and measured at D6 (end of pre-growth step) and D13 (end of growth step), the properties of the bacteria contained in these tubes, i.e., the optical density at 565 nm (OD_565_) as well as the magnetic response (MR) of the bacterial suspensions, which is the percentage of bacteria that orientates parallel to a magnetic field applied by a magnet as observed by optical microscopy. We defined that the minimal growth media (Examples 1–4 of Supplementary Information) that did not weaken bacterial growth and MR were:

- A mineral elixir, which was simplified compared with ME1 in Zhang et al. containing 14 chemicals, i.e., nitrilotriacetic acid, MgSO_4_, MnSO_4_, NaCl, FeSO_4_, CoSO_4_, CaCl_2_, ZnSO_4_, CuSO_4_, KAl(SO_4_)_2_, H_3_BO_3_, Na_2_MoO_4_, NiCl_2_, and Na_2_SeO_3_, to ME6 containing the two chemicals, i.e., FeSO_4_ and CaCl_2_, leading indeed to a larger OD_565_ for ME6 (OD_565_ ~ 0.32 at D6 and OD_565_ ~ 1.52 at D13) than for ME1 (OD_565_ ~ 0.03 at D6 and OD_565_ ~ 0.26 at D13) and similar MR at D13 of 95–100% for ME6 and ME1 (Tables S1, S2, Example 1 of Supplementary Information).
- A yeast extract equivalent, which was simplified compared with a standard yeast extract (YE) in Zhang et al., mainly containing proteins, nucleic acids, functional peptides, volatile aromatic compounds and other components such as trace element, to only one vitamin, i.e., Thiamine HCl, yielding similar OD_565_ (OD_565_ ~ 0.26–0.32 at D6 and OD_565_ ~ 1.1–1.51 at D13) and MR (95–100% at D13) for Thiamine HCl and YE (Tables S3–S5, Example 2 of Supplementary Information).
- K_2_HPO_4_ with a concentration, which was decreased from 0.5 g/L in Zhang et al. to 0.1 g/L, leading to similar OD_565_ for these two concentrations of ~ 0.5–0.7 at D6 and ~ 1.1 at D13, and MR of 95–100% (Tables S6, S7, Example 3 of Supplementary Information).

We deduced from these experiments that the minimal pre-growth and growth media contained a majority, i.e., more than 0.1 g/L, of sodium lactate, NH_4_Cl, MgSO_4_·7H_2_O, K_2_HPO_4_, and a minority, i.e., <0.5 mg/L, of thiamin HCl, ferrous sulfate heptahydrate, and CaCl_2_ (see “*Materials and Methods”* for a detailed composition). This corresponded to a drastic reduction in chemicals compared with the growth media of Zhang et al.

We then optimized the composition of the acidic fed-batch medium using a 1 L fermentation system that mimics the conditions of the fed-batch conditions described in Zhang et al. (details in “*Materials and Methods”*). For that, the growth step started from 800 mL of bacteria originating from the pre-growth step, which were amplified up to an OD ~ 0.1 in the optimized pre-growth medium. Bacterial growth and magnetosome production were triggered by supplying the growth medium with oxygen on the one hand and fed-batch medium on the other hand. As for the pre-growth and growth media, all constituents not compatible with medical regulation (YE, heavy metals apart from iron, and CMR agents) were removed from the fed-batch medium and the concentrations of the remaining elements (lactic acid, ammonia, magnesium sulfate, K_2_HPO_4_ and FeCl_3_·6H_2_O) were each diluted by factors of 2, 5, and 10 compared with Zhang et al. Among all different tested conditions (Table S8), we found that the minimal fed-batch medium that enables maintaining bacterial growth and magnetosome production contained a majority, i.e., more than 0.5 g/L, of L-(+)-Lactic acid, ammonia, K_2_HPO_4_, MgSO_4_·7H_2_O, ferric chloride hexahydrate, and a minority, i.e., <0.5 mg/l, of thiamine hydrochloride, ferrous sulfate heptahydrate, and CaCl_2_ (see “*Materials and Methods”* for a detailed composition). Indeed, after 48 h of growth this minimal fed-batch medium (RFM) led to similar OD_565_ and MR than the non-minimal fed-batch medium (NRFM) of Zhang et al., i.e., OD_565_ ~ 1.6 and MR of 95 % for RFM compared with OD_565_ ~ 1.2 and MR of 95–100% for NRFM (Table S9, Example 4 of Supplementary Information).

We consequently succeeded in maintaining similar MSR-1 growth parameters after adapting the three growth media to pharmaceutical standards by: (i) replacing YE by one vitamin (Thiamine HCl), (ii) removing all voluntary added toxic metals that are usually contained in YE, and (iii) getting rid of the two CMR agents boric acid and nitrilotriacetic acid.

### Magnetotactic Bacteria Grown in Minimal Growth Media

**Figure 1:**
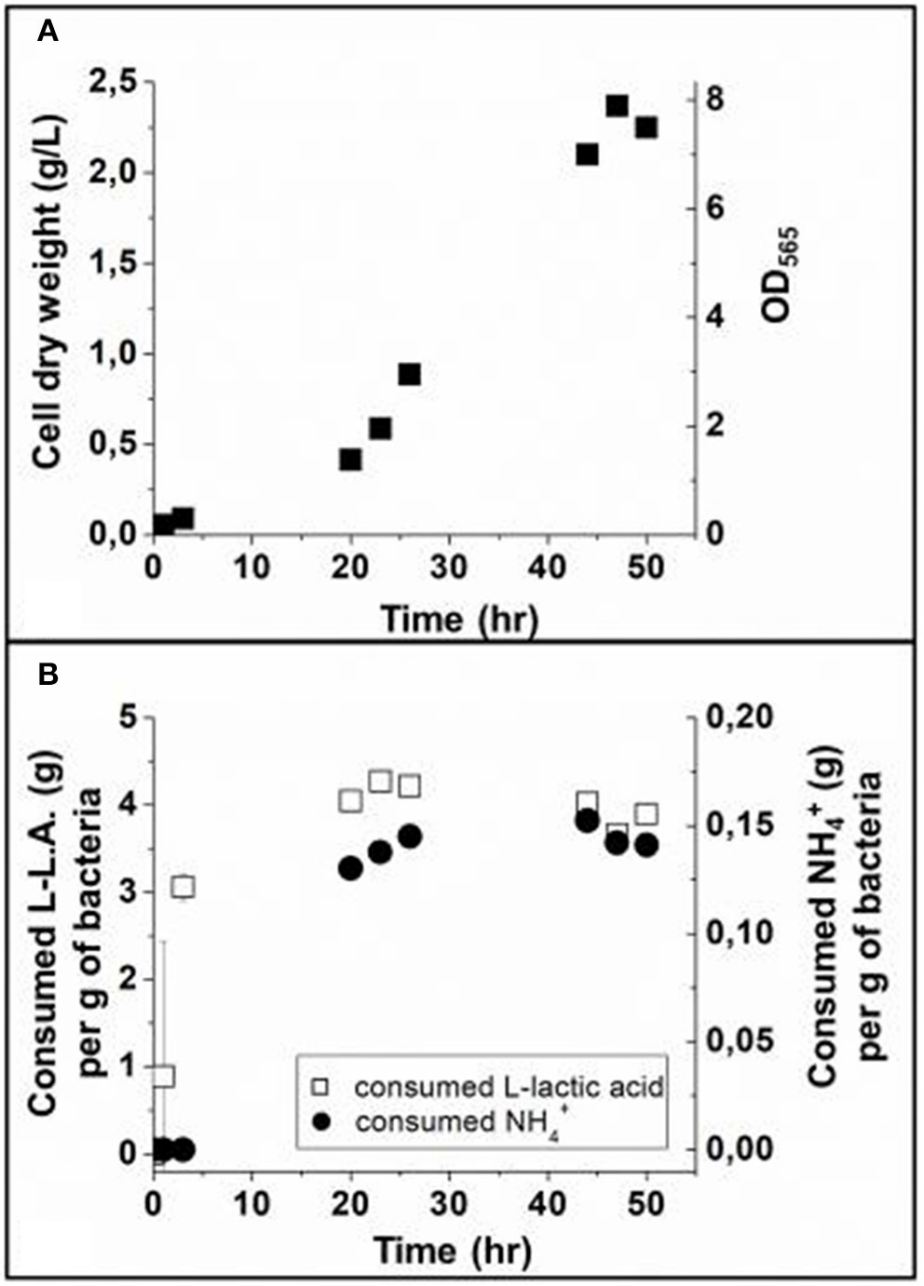
**(A)** Cell growth curve in a 70 L fermenter, represented by the variation of cell dry weight and OD_565_ (measurement error < 5%) as a function of time during the growth step. **(B)** Consumed L lactic acid (L-LA) and ammonium (${\text{NH}}_{4}^{+}$) as a function of time during the growth step, estimated in gram of L-LA per gram of bacteria and gram of ${\text{NH}}_{4}^{+}$ per gram of bacteria. The dissolved O_2_, dO_2_, was maintained between 0.1 and 1% by the regulation of airflow, after which it decreased from 12% at 0 h (T = 0 h) to below 1% at 6 h (T = 6 h). The magnetic response is negative before 20 h (T = 20 h) and positive after. These analyses were performed in triplicate. The error bars represent standard deviations.

Using the three optimized media described in the previous section, we report the results about growth of MSR-1 magnetotactic bacteria. 10 L of these bacteria were pre-amplified in the minimal pre-growth medium up to an OD_565_ ~ 0.4 and were transferred into a 70 L fermenter filled with 30 L of minimal growth medium. Bacterial growth was stimulated by bubbling oxygen in the growth medium while maintaining the dissolved oxygen concentration (dO_2_) of this medium below 1% to allow magnetosome synthesis (Heyen and Schüler, 2003). The growth medium was further supplemented by the minimal acidic iron rich fed-batch medium that kept the pH of the growth medium at 6.9, hence promoting cell division and providing iron for magnetosome synthesis. Growth, which was followed by measuring the variation of cell dry mass as a function of time (Figure 1A), was stopped at 50 h, after reaching stationary phase, i.e., a stable OD_565_ ~ 8. During bacterial growth, we measured the concentrations of L-Lactic acid (L-L.A) and ${\text{NH}}_{4}^{+}$ by high performance liquid chromatography and Nessler colorimetric reaction, respectively, leading to an estimate of the bacterial consumption of these two nutrients (Figure 1B). Although we clearly observed a change in the rhythm of bacterial growth, which was associated with the usual three-phase bacterial growth behavior, it is beyond the scope of this paper to determine the precise duration of each phase as well as the exact time at which the transition from one phase to the other occurs.

During the initial lag phase, i.e., between 0 and ~20 h, Figure 1A shows that bacterial cell population slowly increases by ~0.017 g/L/h, and that the consumption of L-LA increases more rapidly than that of ${\text{NH}}_{4}^{+}$, leading to saturation of L-LA and ${\text{NH}}_{4}^{+}$ consumptions, i.e., 3.06 g per g of bacteria (g/g_B_) at 3–4 h for L-LA compared with 0.15 g/g_B_ at 20 h for ${\text{NH}}_{4}^{+}$. Following the lag phase, Figure 1A shows that the exponential phase occurs between ~20 and ~44 h, being characterized by a much more rapid increase of the dry mass at a mean rate of ~0.07 g/L/h and by larger consumptions of ${\text{NH}}_{4}^{+}$ and L-L.A, which remain at the saturating levels mentioned above. The growth ends by the stationary phase between 47 and 50 h, which results in the saturation of the produced dry mass at 2.25 g/L and to an absence of large variations in ${\text{NH}}_{4}^{+}$ and L-L.A consumptions (Figures 1A,B). The following two aspects deserve to be underlined concerning the link between bacterial growth and L-LA/ ${\text{NH}}_{4}^{+}$ consumption:

On the one hand, exponential bacterial growth directly follows L-LA and ${\text{NH}}_{4}^{+}$ consumption acting as carbon and nitrogen sources, respectively, for magnetotactic bacteria, triggering the synthesis of various biological material (proteins, lipids…) and more generally enabling cell metabolism and especially growth and magnetosome synthesis (Naresh et al., 2012).

On the other hand, the delayed consumption of ${\text{NH}}_{4}^{+}$ compared with L-LA (Figure 1B), suggests that magnetotactic bacteria have a larger need for nitrogen during the exponential than lag phase possibly due the exponential growth requiring an enhanced quantity of proteins for magnetosome vesicle formation and/or transport/incorporation of iron in magnetosomes (Moisescu et al., 2014).

Although bacterial amplification seemed slightly less pronounced than in Zhang et al., we reached similar results in terms of yields than in one of the best other growth protocols described in Sun et al. (2008), i.e., maximum concentration of dried bacteria of 2.4 g_DB_/L (g of bacteria per liter of growth medium) compared with 2.2 g_DB_/L reached after 60 h of cultivation in Sun et al. (2008). This indicates that reduction or removal of uncharacterized products such as animal derived ingredients, CMR products, and metals different from iron did not significantly affect bacterial growth. This is a very interesting and largely unexpected result since metals have been reported to be essential nutrients for the growth of numerous living organism such as bacteria (Boyaval, 1989).

### Production of Magnetosomes

**Figure 2:**
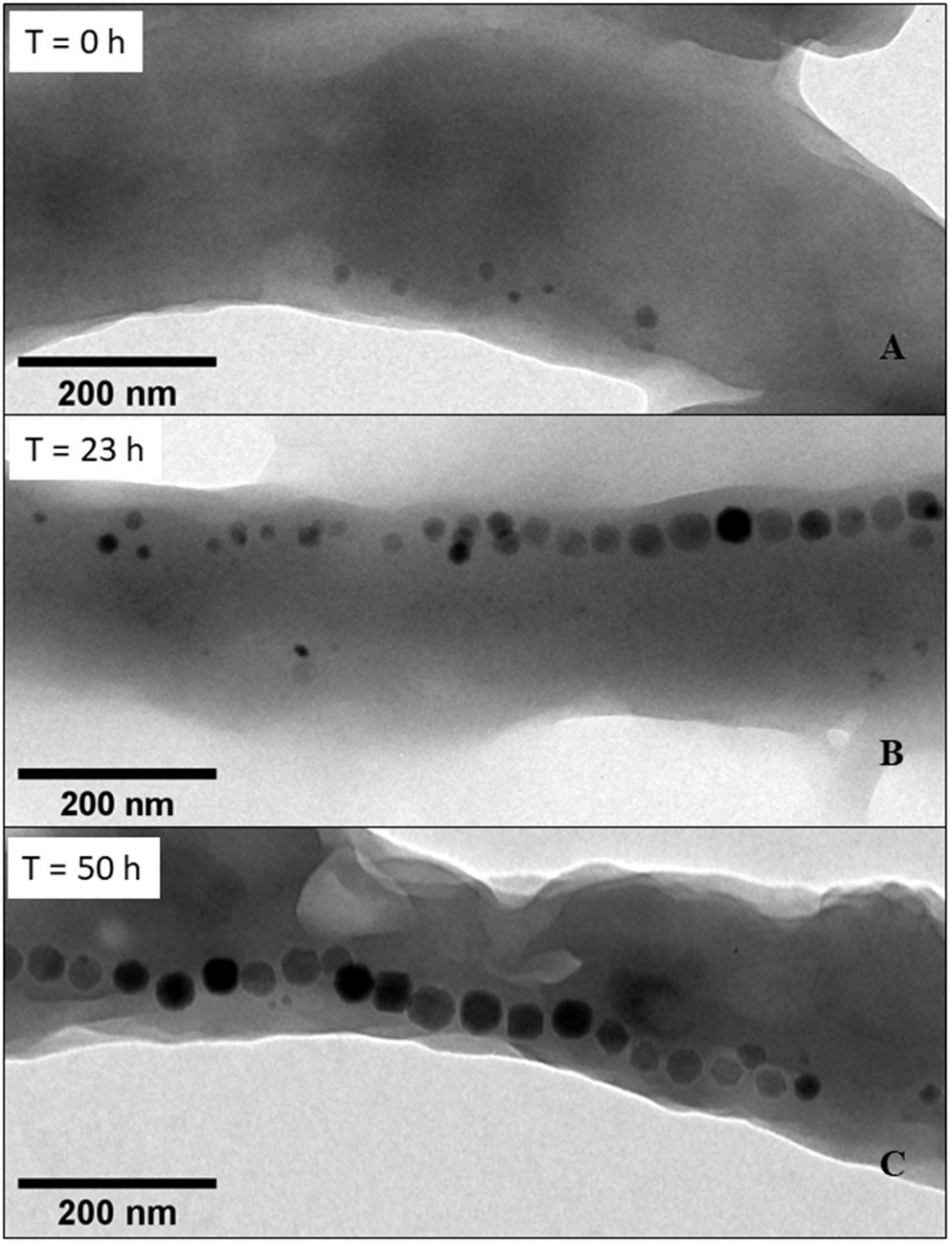
Transmission electron microscopy (TEM) images of a typical magnetotactic bacterium 0 h (T = 0 h), 23 h (T = 23 h), or 50 h (T = 50 h), showing a few individual magnetosomes at T = 0 h, an incompletely formed chain of magnetosomes at T = 23 h, and a fully formed chain of magnetosomes at T = 50 h. To carry out these observations, suspensions containing whole magnetotactic bacteria were deposited and dried on top of a carbon grid and then observed by TEM.

To study the evolution of magnetosome properties as a function of growth time, we examined ~200 magnetotactic bacteria by TEM at different growth time points. At the beginning of bacterial growth (0 h), i.e., before any fed-batch intake, Figure 2A shows that only a few individual magnetosomes are present in MSR-1 magnetotactic bacteria. At 0 h, 62% of magnetotactic bacteria contain isolated magnetosomes while 38% of them lack nanoparticles. The same trends were observed following 1 and 3 h of bacterial growth. Afterwards, we observed the progressive appearance of magnetosome chains, i.e., incomplete chains at 23 h (Figure 2B) and fully formed chains at 50 h (Figure 2C). In addition to magnetosome spatial organization, we measured magnetosome number (n) and diameter (D) per/in bacterium at different time points (0, 1, 3, 20, 23, 26, 44, 47, and 50 h) during the 50 h of bacterial growth (Figures S1A,B). These two parameters increased from *n* = 2.5 ± 2.4 and D = 15.8 ± 4.5 nm at 0 h to *n* = 28.3 ± 7.8 and D = 33.5 ± 6.2 nm at 50 h, reaching a slightly larger value of *n* and smaller value of D compared with *n* ~ 20 and D ~ 42 nm determined for magnetotactic bacteria grown under environmental conditions (Schleifer et al., 1991; Scheffel et al., 2008; Lohße et al., 2016) or using the same methods/media as those of Zhang et al. (Le Fèvre et al., 2017; Mandawala et al., 2017) with D = 37.6 ± 8.8 nm and *n* = 22.8 ± 8.1 after 50 h of cultivation.

### Iron Allocation During Growth

**Figure 3:**
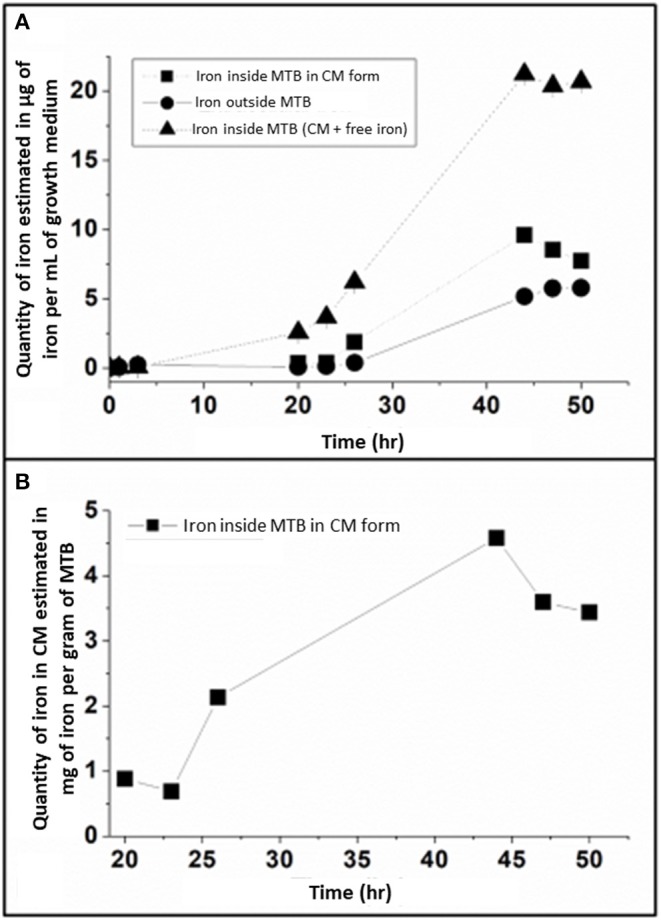
**(A)** Evolution of the repartition of iron during the 50 h of fermentation, which is divided between: (i) iron inside MTB in magnetosome chain form, (ii) iron outside MTB, and (iii) iron inside MTB both in magnetosome chains and other iron forms, where the quantities of the three different types of iron are estimated in μg of iron per mL of growth medium. The error bars represent standard deviations. Standard deviations values are, respectively: 0.005, 0.003, 0.006, 0.014, 0.222, and 0.066 at 20, 23, 26, 44, 47, and 50 h for iron inside MTB under magnetosome chains form; 0.001, 0.003, 0.002, 0.002, 0.010, 0.054, 0.040, 0.105 at 1, 3, 20, 23, 26, 44, 47, and 50 h for iron outside MTB; 1.10^−4^, 1.10^−4^, 1.10^−4^, 0.021, 0.012, 0.041, 0.383, 0.174, 0.179 at 0, 1, 3, 20, 23, 26, 44, 47, and 50 h for iron inside MTB. **(B)**, Variation of the quantity of iron in magnetosome chain (MC) form, estimated in mg of magnetosome chains per gram of MTB, during the 50 h of the growth step. The error bars represent standard deviations. Standard deviations values are, respectively, 0.012, 0.004, 0.006, 0.007, 0.094, 0.029 at 20, 23, 26, 44, 47, and 50 h. T Extracellular iron is estimated in the supernate obtained after centrifugation of whole MTB. Total intracellular iron is obtained by estimating the quantity of iron in the bacterial pellet obtained after centrifugation. The intracellular iron, which is in the crystallized form (magnetosome chains) inside MTB, is obtained by estimating the quantity of iron in magnetosomes extracted from whole MTB, which are obtained following lysis of whole MTB. These analyses were performed in triplicates.

To examine the allocation of iron during growth, we have studied the incorporation of iron both in whole magnetotactic bacteria and in magnetosomes during the 50 h of growth in minimal growth media by measuring the distribution of iron between the interior and exterior of these bacteria and by further dividing total internalized iron between ionic and crystallized iron (Figure 3).

Between 0 and 26 h, Figure 3 shows that extracellular iron concentration (outside bacteria) stays at a very low level of 0–0.4 μg/mL, while total intracellular iron concentration increases from 0.040 ± 0.0004 μg/mL at 0 h to 2.6 ± 0.02, 3.7 ± 0.01, and 6.2 ± 0.04 μg/mL at 20, 23, and 26 h, respectively, indicating that within this period of time, i.e., between 20 and 26 h, 94–97% of iron brought by the fed-batch medium to the growth medium is internalized inside magnetotactic bacteria. By contrast, between 26 and 44 h, Figure 3A shows that intracellular and extracellular iron concentrations both increase more rapidly than between 0 and 26 h to reach a plateau at 44 h of ~21 and ~5 μg/mL, respectively. These behaviors mean that after a certain threshold is reached, bacteria are not able to internalize all iron that is brought to them sufficiently rapidly, leading to an increase of extracellular iron concentration. Finally, at the end of bacterial growth, i.e., between 47 and 50 h when fed-batch medium is not anymore added to the growth medium and bacteria have stopped dividing (Figure 1A), Figure 3A shows that extracellular/intracellular iron concentrations remain unchanged, suggesting that variations in iron allocation follow a dynamic governed by bacterial growth.

Turning to an analysis of the quantity of intracellular iron being incorporated in chains of magnetosomes (Q_FeMC_), it appears that Q_FeMC_ is undetectable before 20 h (Figure 3A), in agreement with the quasi-absence of magnetosome chains in the TEM images of MSR-1 magnetotactic bacteria taken at early growth times, when only small individual magnetosomes are detectable (Figure 2A). Afterwards, Q_FeMC_ increases from 0.9 mg of magnetosome chains per gram of bacteria (μg_MC_/g_B_) at 20 h to 4.6 mg_MC_/g_B_ at 44 h. This behavior can be attributed firstly to a quantity of iron being incorporated in MTB, which increases from 6.2 μg/mL at 20 h to 21.2 μg/mL at 44 h (Figure 3A), and secondly to a percentage of intracellular iron being transformed into magnetosome chains, which increase from 14% at 20 h to 45% at 44 h (Table 1). This percentage of intracellular iron being transformed into magnetosome chains was calculated as follows Q_FeMC_/(Q_FeOB_+Q_FeIB_), where Q_FeOB_ and Q_FeIB_ are the quantities of iron outside and inside MTB, respectively.

**Table 1:** Percentage of intracellular iron under magnetosome chains (MC) and associated standard deviations at different time points of the growth step, i.e., 20, 23, 26, 44, 47, and 50 h following the beginning of the growth step.

**Time (h)**	**20**	**23**	**26**	**44**	**47**	**50**
Intracellular iron under MC form (%)	14.1 (±0.20)	11.0 (±0.07)	30.4 (±0.09)	45.3 (±0.06)	41.8 (±1.10)	37.4 (±0.32)

### Distribution in Magnetosome Composition Between Iron and Other Metals

**Figure 4:**
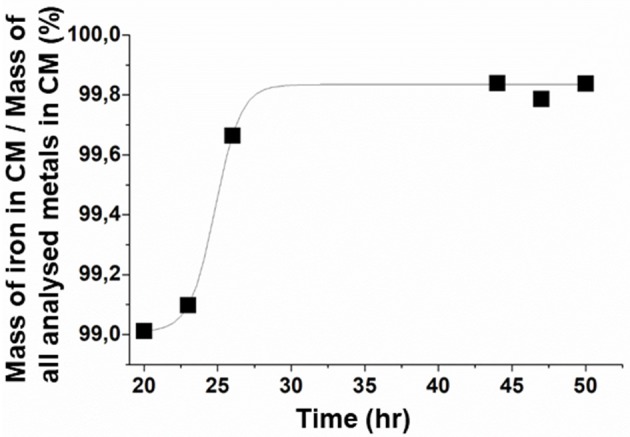
Evolution during the course of the growth step of the percentage in mass of iron in magnetosome chains (MC) relatively to the mass of all analyzed metals (Ag, Al, As, Ba, Cd, Co, Cr, Cu, Fe, Mn, Mo, Ni, Pb, Sb, Se, Si, Ti, Tl, W, Zn) in magnetosome chains. These analyses were performed in triplicates. The error bars represent standard deviations. Standard deviations values are, respectively, 0.016, 0.009, 0.002, 7.10^−4^, 0.001, 4.10^−4^ at 20, 23, 26, 44, 47, and 50 h.

Next, to evaluate the “relatve purity” of magnetosome composition in iron relatively to other metals, we determined for magnetosome chains the percentage in mass of iron mass relatively to the mass of a large number of other metals at different growth times, i.e., at 20, 23, 26, 44, 47, and 50 h. At 20 h, this percentage, which can be expressed as P_Fe/i_ = M_Fe_/ΣM_i_, where M_Fe_ is the mass of iron in magnetosome chains and M_i_ is the mass of the various analyzed metals (M_Ag_, M_Al_, M_As_, M_Ba_, M_Cd_, M_Co_, M_Cr_, M_Cu_, M_Fe_, M_Mn_, M_Mo_, M_Ni_, M_Pb_, M_Sb_, M_Se_, M_Si_, M_Ti_, M_Tl_, M_W_, M_Zn_) in magnetosome chains, is *P* ~ 99%. *P*, which is already quite large at 20 h, increases even more to reach a plateau at 44 h of ~99.8%, remaining at this value until the end of growth, i.e., *P* ~ 99.8% at 50 h (Figure 4). Such improvement in magnetosome chain relative purity between 20 and 50 h could be explained by the increase of the quantity of iron being incorporated in magnetosome chains from 0.9 mg/g of bacteria at 20 h to 3.5 mg/g of bacteria at 50 h (Figure 3B) and by the quantity of other metals than iron being incorporated in magnetosome chains that remains relatively unvaried at ~6–8 μg/g of bacteria between 20 and 50 h and which is ~100–500 times lower than the quantity of incorporated iron (Table 2). A finer analysis of the decrease in the proportion of metals other than iron in magnetosome chain composition during MSR-1 growth reveals essentially two types of behavior depending on metals (Table 3):

- For the four most abundant metals at 20 h, i.e., Ba, Cr, Ni, Ti, their concentration estimated in μg per gram of iron in magnetosome chains progressively decreases until the end of exponential phase and then remains stable, i.e., Ba decreases from 1,292 μg/g_Fe_ at 20 h to 351 μg/g_Fe_ at 50 h, Cr concentration decreases from 1583 μg/g_Fe_ at 20 h to 106 μg/g_Fe_ at 50 h for Cr, Ni concentration decreases from 5,036 μg/g_Fe_ at 20 h to 436 μg/g_Fe_ at 50 h, and Ti concentration decreases from 930 μg/g_Fe_ at 20 h to 140 μg/g_Fe_ at 50 h.
- For the less abundant metals at 20 h, i.e., Ag, Al, As, Cd, Co, Cu, Mn, Mo, Pb, Sb, Se, Si, Tl, W, Zn, their concentration is too low at 20 h to be able to observe a clear tendency concerning their variation in quantity.

**Table 2:** Evolution of the quantity of iron and metals different from iron, estimated in μg of iron or metals other than iron in magnetosome chains (MC) per gram of bacteria, at different time points of the growth step, i.e., 20, 23, 26, 44, 47, and 50 h following the beginning of the growth step and associated standard deviations.

**Time (h)**	**20**	**23**	**26**	**44**	**47**	**50**
MC iron produced (μg) per g of bacteria (μgCM/gB)	879.0 (± 12.3)	686.5 (± 4.3)	2131.1 (± 6.4)	4577.8 (± 6.6)	3595.0 (± 93.8)	3435.9 (± 29.4)
Heavy metals exept iron inMC (μg) per g of bacteria (μgCM/gB)	8.2 (± 0.2)	5.6 (± 0.4)	7.2 (± 0.2)	7.2 (± 0.1)	8.2 (± 0.1)	5.7 (± 0.1)

**Table 3:** Evolution of the quantity of metals other than iron (Ag, Al, As, Ba, Cd, Co, Cr, Cu, Mn, Mo, Ni, Pb, Sb, Se, Si, Ti, Tl, W, Zn) at different time points of the growth step, i.e., 20, 23, 26, 44, 47, and 50 h following the beginning of the growth step, estimated in μg of metals others than iron per gram of iron in magnetosome chains (MC).

**CM obtained with minimal growth media**							**CM obtained with non-minimal growth media**
Element	T (20 h)	T (23 h)	T (26 h)	T (44 h)	T (47 h)	T (50 h)	Zhang et al., 2011 T(50 h)
Ag	ND	ND	ND	ND	ND	ND	34
Al	286 (65%)	440 (100%)	226 (51%)	405 (92%)	250 (57%)	306 (70%)	1,308
As	ND	ND	ND	ND	ND	ND	ND
Ba	1,292 (100%)	740 (57%)	669 (52%)	354 (27%)	384 (30%)	351 (27%)	9,903
Cd	18 (65%)	15 (53%)	20 (72%)	24 (86%)	28 (100%)	23 (83%)	25
Co	ND	87 (100%)	7 (8%)	ND	ND	ND	48
Cr	1,583 (100%)	1,548 (98%)	400 (25%)	113 (7%)	117 (7%)	106 (7%)	421
Cu	ND	ND	ND	ND	ND	ND	155
Mn	ND	ND	ND	ND	ND	ND	50,140
Mo	ND	ND	ND	ND	ND	ND	16
Ni	5,036 (100%)	5,005 (98%)	1,815 (36%)	403 (8%)	473 (9%)	436 (9%)	671
Pb	ND	26 (26%)	99 (100%)	47 (47%)	53 (54%)	21 (22%)	123
Sb	ND	ND	ND	ND	ND	ND	ND
Se	ND	ND	ND	ND	ND	ND	ND
Si	642 (67%)	963 (100%)	ND	5 (1%)	135 (14%)	138 (14%)	ND
Ti	930 (100%)	280 (30%)	137 (15%)	134 (14%)	163 (18%)	140 (15%)	236
Tl	192 (100%)	ND	ND	16 (8%)	24 (12%)	33 (17%)	72
W	ND	ND	ND	ND	ND	ND	ND
Zn	ND	ND	ND	111 (22%)	504 (100%)	64 (13%)	1204
Total	9,979	9,104	3,373	1,613	2,131	1,620	64,356

*These analyses were performed in triplicates. Standard deviation, respectively: Al: 267, 72, 3, 8 and 2 at 20, 23, 26, 44, 47, and 50 h*.

*Ba: 37, 24, 5, 1, 12 and 3 at 20, 23, 26, 44, 47, and 50 h*.

*Cd: 4, 14, 4, 0, 0 and 0 at 20, 23, 26, 44, 47, and 50 h*.

*Co: 64 and 13 at 23 and 26 h*.

*Cr: 68, 89, 11, 4, 1 and 2 at 20, 23, 26, 44, 47, and 50 h*.

*Ni: 95, 82, 19, 3, 21 and 1.6 at 20, 23, 26, 44, 47, and 50 h*.

*Pb: 414, 43, 19, 6 and 1 at 23, 26, 44, 47, and 50 h*.

*Si: 400, 133, 21, 44 and 5 at 20, 23, 44, 47, and 50 h*.

*Ti: 35, 18, 8, 1, 6 and 1 at 20, 23, 26, 44, 47, and 50 h*.

*Tl: 556, 17, 11 and 16 at 20, 44, 47, and 50 h*.

*Zn: 4, 19 and 4 at 44, 47, and 50 h*.

*ND: Not detected*.

The efficacy of our method is further highlighted by the value of *P*, which is much larger when magnetosome chains were synthesized in minimal growth media, i.e., *P* ~ 99.8%, than when they were produced using the non-minimal growth media of Zhang et al., i.e., *P* ~ 93.6%, Table 3.

## Discussion

Here, we show for the first time that it is possible to grow MTB in minimal growth media that are devoid of yeast extract, heavy metals different from iron, and CMR agents, following a growth method in two steps, where MTB were first amplified in a pre-growth-step without producing magnetosomes, and were then fed with an iron-rich fed-batch medium in a growth step to allow magnetosome synthesis, resulting after 50 h of growth in an OD_565_ ~ 8 and a good yield of magnetosome production, i.e., ~10 mg of magnetosomes per liter of growth medium. These results are surprising since an important number of metals have been identified as being essential for bacterial growth, i.e., Na, K, Mg, Ca, V, Cr, Mn, Fe, Co, Ni, Cu, Zn, Mo, and Se (Boyaval, 1989). It is usually recommended to add certain metals to bacterial growth media since such metals are known to play different roles such as acting as cofactors of certain anti-oxidant enzymes or enabling glycosylation for Mn (Dürr et al., 1998). In the present study, the residual essential metal contents contained in the reagents of the minimal media have been sufficient for fulfilling the metabolic requirements of the MSR-1 strain. Indeed, trace amount of metals like Mn, Fe, Cu, Zn, and Mo have been reported to be sufficient for bacterial growth (Hughes and Poole, 1991).

While during the majority of the growth, MTB consume carbon and nitrogen, they only divide and produce magnetosomes significantly after 20 h when the quantity of iron brought to the growth medium becomes significant in micro-aerobic conditions, making the combination of the three sources (carbon, nitrogen and iron) essential for MTB activity, a finding that can seem obvious given the function of MTB but that was not previously reported before as clearly as in this study, since the role of the iron source was not disentangled from that of the carbon and nitrogen sources (Naresh et al., 2012).

The rate of magnetosome chain formation (MCF) shows three phases, divided between an absence of MCF between 0 and 20 h, significant MCF between 20 and 44 h, and saturating MCF beyond 44 h, these three phases occurring within the same periods of times as the lag, exponential, and stationary phases of bacterial growth, suggesting that enhanced magnetosome production is correlated with cellular division. Therefore, MTB do not lose their faculty to produce magnetosomes when they divide, suggesting that cellular division is not accompanied by MTB genetic modifications, in particular removals of the genes responsible for magnetosome synthesis (Schübbe et al., 2003).

Interestingly, we also observed that only a minority of intracellular iron was in a crystallized form, suggesting that the majority of intra-cellular iron is not used for magnetosome synthesis. Over the whole growth, the maximum percentage of intracellular iron transformed in magnetosome chains (45%) is similar to that of 50% reported in one study (Amor et al., 2018) and lower than that of 99.5% reported in two other studies (Grünberg et al., 2004), suggesting that as in Amor et al., iron does not only serve for magnetosome formation but also for the overall metabolic activity of MTB (Naresh et al., 2012) or is stored in cells under other forms than magnetite (Amor et al., 2018). Furthermore, it is possible that the different results reported in Grünberg et al. (2004) than in this study or Amor et al. (2018) is due to the quantity of available iron in MTB growth medium, which is more important here and in Amor et al. (2018) than in Grünberg et al. (2004).

We have also studied the impact of minimal growth media on magnetosome composition. Whereas, we increased magnetosome iron relative purity by reducing the concentration of other metals than iron in the growth media, i.e., the concentration of iron relatively to other analyzed metals in magnetosomes increases from 93.8% using non-minimal growth media (Zhang et al., 2011) to 99.8% using minimal growth media, previous studies have reported a faculty of MTB to incorporate other metals than iron, such as Co, Zn or Ni, in magnetosomes by growing MTB in media enriched with these metals (Staniland et al., 2008; Kundu et al., 2009; Li et al., 2016). Another interesting observation lies in the rate of metal incorporation, which seems to depend on the type of metal that is considered. Among the different metals, Mn appears to reach one of the largest incorporation in magnetosome crystal structure, i.e., 5% for MTB cultivated in non-minimal growth media (Table 1), which is close to the values of 1–2.8% of incorporated Mn relatively to total content of analyzed metals in magnetosomes (Keim et al., 2009; Tanaka et al., 2012; Prozorov et al., 2014). In the non-minimal growth media, Mn was the preponderant incorporated metal since it corresponded to 78% in mass of all analyzed metals different from iron incorporated in magnetosomes (Table 1). To the author knowledge, the only other element that could be incorporated more importantly than Mn was Cu as demonstrated for the AMB-1 strain grown in a specific medium containing a large Cu concentration that was close to the toxicity threshold, leading to 15% of Co in the magnetosomes (Tanaka et al., 2012). Interestingly, although certain metals such as Mn, Cu, and Co have a tendency to be incorporated in magnetosomes (Tanaka et al., 2012), they are undetectable in magnetosomes produced with minimal growth media and therefore do not seem essential to magnetosome synthesis. As a whole, these different studies including ours indicate that it is possible to tune magnetosome composition, either toward an iron-richer or an iron more depleted composition by adjusting the proportion of metals different from iron relatively to iron within MTB growth medium composition.

Finally, we examine how the composition of the growth medium influences magnetosome sizes and magnetosome number per bacteria. Our study combined with other results presented elsewhere leads to the conclusion that a minimal medium with very low concentrations of metals other than iron results in a change in magnetosome diameter from 37.6 ± 8.8 nm for MTB grown in non-minimal growth media (Le Fèvre et al., 2017), to 33.5 ± 6.2 nm for MTB amplified in minimal growth media (Figure S1A), and change of magnetosome number in magnetosome chains from 22.8 ± 8.1 for MTB grown in non-minimal growth media (Le Fèvre et al., 2017), to 28.3 ± 7.8 for MTB amplified in minimal growth media (Figure S1B). Those changes appear to be statistically not significant. However, changes in average values could be linked by the absence of certain metals such as Co, Ni, and Zn that are known to yield an increase in magnetosome sizes (Kundu et al., 2009). For example, a medium that is intentionally enriched with Co below a toxic threshold results in an increase of magnetosome sizes for three different strains of MTB, i.e., from 45.5 ± 18.3 nm to 47.3 ± 6.8 nm for MSR-1, from 39.8 ± 10.0 nm to 43.8 ± 9.9 nm for MS-1, and from 52.6 ± 16.0 nm to 58.2 ± 11.7 nm for AMB-1 between before and after Co enrichment (Staniland et al., 2008). We therefore acknowledge that the composition of MTB growth media might affect magnetosome sizes and numbers but that these changes as reported in the present study are rather small. Figures S1A,B, which present TEM images of magnetosomes extracted from whole MTB produced in minimal and non-minimal growth media, respectively, showing in both cases magnetosomes with a homogenous distribution, indicating that the dispersity quality has not been lost by using minimal growth media.

## Materials and Methods

### MTB Bacterial Strain

*Magnetospirillum gryphiswaldense* strain MSR-1 (DSM6361) was purchased from Deutsche Sammlung von Mikro-organismen und Zellkulturen (Brunswick, Germany) and stored in small aliquots of 1 mL at −80°C.

### Composition of Optimized Minimal Growth Media of MTB

Minimal pre-growth and growth media were determined in 50 mL tubes while minimal fed-batch medium was determined in 1.5 L fermenter.

Minimal pre-growth medium contained in 1 L of water: 2.6 g of Na-lactate, 0.4 g of NH_4_CL, 0.1 g of MgSO_4_·7H_2_O, 0.1 g of K_2_HPO_4_, 4·10^−5^ g of Thiamine. HCL, and 5·10^−4^ g of FeSO_4_·7H_2_O, and 0.01 g of CaCl_2_·2H_2_O.

Minimal growth medium contained in 1 L of water: 1.3 g of Na-lactate, 0.22 g of NH_4_CL, 0.027 g of MgSO_4_·7H_2_O, 0.027 g of K_2_HPO_4_, 4·10^−5^ g of Thiamine.HCL, and 8·10^−5^ g of FeSO_4_·7H_2_O, and 1.6·10^−3^ g of CaCl_2_·2H_2_O.

Minimal fed-batch medium contained in 1 L of water: 100 g of lactate, 4.8 g of NH_3_, 0.48 g of MgSO_4_·7H_2_O, 3 g of K_2_HPO_4_, 0.4 g of Thiamine.HCL, 2 g of FeCl_3_·6H_2_0, 7·10^−3^ g of FeSO_4_·7H_2_O, and 0.14 g of CaCl_2_·2H_2_O.

All chemicals were pharmaceutical grade (Merck).

### Culture of MTB in 50 ml Tubes

During a first step, cryo-stocks of MSR-1 bacteria were grown in 50 mL tubes filled with 8 mL of pre-growth medium and incubated during 6 days until D6 at 29.5°C in an orbital shaker incubator operated at 100 rpm. During a second step, 50 mL tubes were filled with 30 mL of growth medium supplemented with iron citrate (at a concentration of 200 μM) acting as the iron source and incubated during 7 days until D13 in the same conditions as in the first step of culture. Samples of 2 mL were taken at the end of each step of culture (D6 and D13) to determine the optical density at 565 nm, OD_565_, at D6 and D13, and the magnetic response of the bacteria, MR, at D13. This experiment was performed in triplicate.

### Culture of MTB in 1.5 L Bioreactor

During a first step, cryo-stocks of MSR-1 bacteria were grown in 500 mL bottles filled with 250 mL of minimal pre-growth medium and incubated during 6 days at 29.5°C in an orbital shaker incubator operating at 100 rpm. During a second step, cells were transferred in DASGIP Parallel 1.5 L Bioreactor System filled with 800 mL of minimal growth medium. Fermentation was carried out at 29.5°C under agitation at 200 rpm during 2 days at pH maintained at 6.9 by adding an acidic fed-batch medium containing FeCl_3_.6H_2_O as the iron source. Growth of MSR-1 bacteria was stimulated by bubbling oxygen in the growth medium at a percentage of dO_2_ kept below 1 % to enable magnetosome synthesis (Sun et al., 2008; Zhang et al., 2011; Yang et al., 2013; Fernández-Castané et al., 2018). Temperature, agitation speed, pH, feeding pump flow and oxygen concentration were monitored and adjusted using a DASGIP controller and a DASware software from Applikon Eppendorf. Samples of 2 mL were taken at 0, 24, and 48 h of the second step of culture. This experiment was performed in triplicate.

### Scale-Up MTB Culture in 70 L Semi-automated Bioreactor

During a first step, cryo-stocks of MSR-1 bacteria were grown in 500 mL bottles filled with 250 mL of minimal pre-growth medium and incubated during 6 days at 29.5°C in an orbital shaker incubator operating at 100 rpm. During a second step, cells were transferred in 10 L bottles filled with 5 L of minimal pre-growth medium and incubated during 2 days at 29.5°C in an orbital shaker incubator operating at 100 rpm. During a third step, cells were transferred in Applikon 70 L fermenter System filled with 40 L of minimal growth medium. Fermentation was carried out in the same conditions as for the 1.5 L fermenters culture. Temperature, agitation speed, pH, feeding pump flow and oxygen concentration were monitored and adjusted using an EZ controller and a BioXpert software from Applikon Biotechnology. Samples of 200 ml were taken at 0, 1, 3, 10, 23, 26, 44, 47, and 50 h of the third step of culture. This experiment was performed in duplicate.

### Sample Preparation

After sampling, cell density (OD_565nm_) and magnetic response were immediately measured. Three different types of samples were then prepared; sample supernatants without cells, whole MSR-1 bacteria (cell pellet) and magnetosome chains extracted from MSR-1 bacteria. For the culture in 70 L fermenter, each culture samples were immediately centrifugated at 4,000 rpm during 30 min. Sample supernatants were separated from the cell pellets and stored in small aliquots at −80°C for further treatment. Cell pellets were washed two times in deionized water by centrifugation at 4,000 rpm during 30 min and stored in aliquots at −80°C for further treatment. To extract the chain of magnetosomes, 40 mL of culture samples were centrifugated at 4,000 rpm during 30 min in 50 mL tubes. Cell pellets were washed two times in deionized water by centrifugation at 4,000 rpm during 30 min and resuspended in 2 M KOH solution at 80°C during 1 h. Magnetosome chains were magnetically separated from organic material residues. The supernatant containing cell debris and other organic material was removed. Magnetosome chains were washed 2 times with PBS 10X and 3 times in deionized water by magnetic selection. Magnetosome chains were then dried at room temperature overnight after the last wash and stored at −80°C for further treatment.

### Measurement of the Cell Density (OD_565nm_) and Magnetic Response

The OD_565nm_ was measured with a spectrophotometer (UviLine9400, SECOMAM, France, linearity of the response for OD_595_ <1) with an OD_565nm_ = 1 corresponded to 0.30 g dry weight L^−1^ (Sun et al., 2008). This dry weight value has been verified with the same protocol as Sun et al. (2008) at 44 h (0.31 g dry weight L^−1^), 47 h (0.29 g dry weight L^−1^), and 50 h (0.28 g dry weight L^−1^) for a final result of 0.29 ± 0.02 g dry weight L^−1^. Optical densities above 1 were estimated by first diluting the sample so that the OD of the diluted sample is below 1, and by then multiplying the OD of the diluted sample by the dilution factor. An Optical microscope and a parallelepipedic neodymium magnet (40 × 10 × 10 mm) were used to evaluate the magnetic response of MSR-1 bacteria. Briefly, MSR-1 bacteria were diluted in deionized water to an OD_565nm_ of 0.25 and dropped on a thin blade for optical microscope observation. The magnet was then manually put successively parallel (position 1) or perpendicular (position 2) with respect to the thin blade containing the suspension of MSR-1 bacteria. Magnetic cells (with magnetosomes) align in the same direction than the magnetic field while non-magnetic or poor magnetic cells (without magnetosomes) are randomly oriented. Images were acquired in position 1 and position 2 with an optical microscope Zeiss Primo Vert (magnification X100) and processed using Zen 2011 lite Zeiss software in auto exposure mode. The magnetic response is calculated by dividing the number of cells (*n* = 200) aligned in positions 1 and 2 by the total number of cells. The magnetic response is positive when the mean orientation of cells is higher than 95%. Below 95%, the magnetic response is negative.

### Transmission Electron Microscopy (TEM)

Cell pellets were thawed at room temperature and washed two times in deionized water by centrifugation at 14,500 rpm during 10 min. Five microliters of each bacterial sample were deposited on top of a carbon-coated copper grid (oxford instruments) and dried. Images were recorded using a JEOL LaB6 JEM-2100 at 200 KeV. TEM images were analyzed with “ImageJ” in order to determine the size of magnetosomes and their amount per cell.

### Lactate and ${\text{NH}}_{4}^{+}$ Assays

Sample supernatants were thawed at room temperature and filtered with a 0.22 μm PES membrane (Millipore). Lactate concentrations were measured by high-performance liquid chromatography (Prominence HPLC, Shimadzu). One milliliter of each sample supernatant was placed with a syringe in the sampler holder and 20 μL of the sample was manually injected into the column. The column used was a Fast Fruit Juice (Waters) and H_3_PO_4_ 5 mM at 0.8 mL/min was used as the eluent. The temperature of the column was maintained at 55°C with a water bath and the separated compound was detected with a refractive index detector (IOTA). Lactate known concentrations were analyzed by HPLC to produce a standard calibration curve in the same operated conditions as for sample supernatants. ${\text{NH}}_{4}^{+}$ concentrations were measured by using the Nessler colorimetric method. One milliliter of each sample supernatant was mixed with 1 ml of arabic gum (0.5% (w/v) and 100 μL of Nessler reagent (potasium iodomercurate and ammonia). Absorbance was read at 400 nm in a spectrophotometer (UviLine9400, SECOMAM, France) after an incubation time of 5 min. ${\text{NH}}_{4}^{+}$ known concentrations were analyzed as well by the Nessler colorimetric method to produce a standard calibration curve in the same operated conditions as for sample supernatants.

### Chemical Analysis

Iron and other metals (Ag, Al, As, Ba, Cd, Co, Cr, Cu, Mn, Mo, Ni, Pb, Sb, Se, Si, Ti, Tl, W, Zn) measurements were made using an inductively coupled plasma atomic emission spectrometer ICP-AES (Thermo Scientific iCAP 6200). For iron and other metals analysis, sample supernatants, MSR-1 bacteria (cell pellets) and extracted magnetosome chains were dissolved in regia solution [70% w/v HNO_3_ and 35% w/v HCl, in a ration 1:3 (v/v)]. The samples were incubated at room temperature overnight in order to dissolve the iron and the other metal species. Ten milliliters of 70% w/v HNO_3_ were added to 10–200 μL of acidified samples considering dilutions of the samples before ICP-AES measurements.

## Conclusion

In this study, we have first developed different minimal growth media enabling the growth of MTB in two steps: (i) a pre-growth step during which MTB are amplified in a pre-growth medium without a large iron concentration, and (ii) a growth step in which MTB are amplified in a growth medium supplemented by an iron-rich fed-batch medium to produce magnetosomes, where the pre-growth, growth, and fed-batch media are depleted in metals different from iron, devoid of yeast extract and CMR products, and contain:

- For the pre-growth medium, 2.6 g of sodium lactate, 0.4 g of NH_4_Cl, 0.1 g of MgSO_4_·7H_2_O, 0.1 g of K_2_HPO_4_, 40 μg of thiamin HCl, and 0.5 mL of ME6.
- For the growth medium, 1.3 g of sodium lactate, 0.2 g of NH_4_Cl, 0.03 g of MgSO_4_·7H_2_O, 0.03 g of K_2_HPO_4_, 27 μg of thiamin HCl, and 0.08 mL of ME6.
- For the fed-batch medium, 100 g of L-(+)-Lactic acid, 4.8 g of NH_3_, 3 g of K_2_HPO_4_, 0.5 g of MgSO_4_·7H_2_O, 2 g of FeCl_3_·6H_2_O, 40 μg of Thiamine HCl and 7 mL of ME6.

where ME6 contains in 1 L of water 1 g of FeSO_4_·7H_2_O and 20 mg of CaCl_2_·2H_2_O.

Theses growth media have a very significantly reduced number of chemicals, i.e., by a factor of ~4, compared with previously used ones (Zhang et al., 2011).

The growth step of MTB displays three phases: (i) the lag phase between 0 and 20 h, where bacteria divide slowly, i.e., the bacterial dry mass (BDM) increases by 0.017 g/L/h, (ii) the exponential phase between 20 and 44 h, where MTB amplify more rapidly at a rate of 0.07 g/L/h in BDM, and (iii) the stationary phase between 47–50 h, where the BDM saturates at 2.25 g/L. The production of magnetosome chains mainly occurs during the exponential phase, where it increases from 0.9 to 4 mg of iron in magnetosome chains per gram of bacteria between the beginning and the end of this phase. Furthermore, during this lapse of time, MTB consume the largest quantity of the carbon (L-Lactic acid) and nitrogen (${\text{NH}}_{4}^{+}$) sources, i.e., 3.06 g of L-LA per gram of bacteria and 0.15 g of ${\text{NH}}_{4}^{+}$ per gram of bacteria. We deduced from these results a direct correlation between magnetosome production and consumption by MTB of the essential nutrients provided by the carbon, nitrogen and iron sources.

Using minimal growth media, we were able to produce “highly pure magnetosomes,” i.e., magnetosomes containing a percentage in mass of iron relative to the other metals analyzed in this study of 99.8% at the end of the growth step. The method that we have implemented to reduce the level of impurities in magnetosomes enables to foresee an optimized safety for use of these nanoparticles in medical applications.

## Data Availability Statement

All datasets generated for this study are included in the article/Supplementary Material.

## Author Contributions

CB, RL, CG, and ER carried out the experiments presented in this study. The main experimental contribution comes from CB. FG, KB, and NB participated in the overall supervision of the research presented in this study. CB and EA wrote most of the MS taking into consideration some advice given by FG, NB, and KB. CB is a PhD student (CIFRE 2014/0747). The research was directed by EA, who is the scientific adviser of the start-up Nanobacterie.

### Conflict of Interest

EA, RL, CB, ER, and CG have been working in the start-up Nanobacterie. The remaining authors declare that the research was conducted in the absence of any commercial or financial relationships that could be construed as a potential conflict of interest.

## Abbreviations

CaCl_2_: Calcium chloride
D: diameter of magnetosomes in bacterium
IONP: iron oxide nanoparticles
FeCl_3_·6H_2_O: Iron chloride hexa-hydrate
K_2_HPO_4_: Potassium phosphate dibasic
L-LA: L-Lactic acid
MC: Magnetosome chains
ME: mineral elixir
MgSO_4_·7H_2_O: Magnesium sulfate hepta-hydrate
MR: magnetic response
MSR-1: *Magnetospirillum Gryphiswaldense*
MTB: magnetotactic bacteria
n: number of magnetosomes per bacterium
NH_3_: Ammonia
${\text{NH}}_{4}^{+}$: Ammonium
NH_4_Cl: Ammonium chloride
OD_565_: optical density measured at 565 nm
TEM: transmission electron microscopy
YE: yeast extract
dO_2_: dissolved oxygen concentration.
