# Supplementary material for: A Method for Producing Highly Pure Magnetosomes in Large Quantity for Medical Applications Using Magnetospirillum gryphiswaldense MSR-1 Magnetotactic Bacteria Amplified in Minimal Growth Media
Source: Front Bioeng Biotechnol. 2020 Feb 18;8:16. doi: 10.3389/fbioe.2020.00016 (PMC7041420; doi:10.3389/fbioe.2020.00016)
Supplement: Supplementary file 1 [file Data_Sheet_1.docx]

**SUPPLEMENTARY MATERIAL**

1. **EXAMPLES:**

**Example 1: Determination of the minimum mineral elixir enabling bacterial growth and magnetosome synthesis:**

In this example non-pharmaceutical grade chemicals were used for the preparation of the growth media. The composition of 1 liter of pre-growth and growth media used for the determination of the minimal mineral elixir are described in *Zhang et al. 2011*. As a whole, 6 different culture conditions were tested corresponding to the 6 different mineral elixirs tested. During a first step of the experiment (D1), cryo-stocks of MSR-1 bacteria were grown in 50 mL tubes filled with 8 mL of pre-growth medium and incubated during 6 days until D6 at 29.5°C in an orbital shaker incubator operated at 100 rpm. During a second step, 50 mL tubes were filled with 30 mL of growth medium supplemented with iron citrate (at a concentration of 200µM) acting as the iron source and incubated during 7 days until D13 in the same conditions as in the first step of culture. Samples of 2 mL were taken at the end of each step of culture (D6 and D13) to determine the optical density at 565 nm, OD_565_, at D6 and D13, and the magnetic response of the bacteria, MR, at D13. A positive magnetic response at D13 and a ratio between the optical density at D13 and the optical density at D6 larger than 4 was observed for conditions ME1, ME3, ME4, ME5 and ME6. In contrast, no magnetic response was observed for condition ME2, where all chemical elements that comprise the mineral elixir where removed. In conclusion, the minimal mineral elixir that enables the growth of MSR-1 bacteria with a large increase in optical density (OD_565nmD13_/ OD_565nmD6_ larger than 4.8) and the synthesis of magnetosomes (positive magnetic response) is ME6, composed only of iron sulfate heptahydrate at a concentration of 1 g/L and of calcium chloride at a concentration of 20 g/L(Table S2).

**Example 2: Determination of a growth medium without yeast extract, enabling the growth of magnetotactic bacteria and the synthesis of magnetosomes:**

In this example we used non-pharmaceutical grade chemicals for the preparation of the growth media. The compositions of pre-growth and growth media, in one liter of de-ionized water, are described in *Zhang et al. 2011*.As a whole, 18 different culture conditions were tested as substitute of the yeast extract (table S3 and Table S4). During a first step of the experiment (D1), cryo-stocks of MSR-1 bacteria were grown in 50 mL tubes filled with 8 mL of pre-growth medium and incubated during 6 days until D6 at 29.5°C in an orbital shaker incubator operated at 100 rpm. During a second step, 50 mL tubes were filled with 30 mL of growth medium supplemented with iron citrate (at a concentration of 200µM) acting as the iron source and incubated during 7 days until D13 in the same conditions as in the first step of culture. Samples of 2 mL were taken at the end of each step of culture (D6 and D13) to determine the optical density at 565 nm, OD_565_, at D6 and D13, and the magnetic response of the bacteria, MR, at D13. A positive magnetic response at D13 and a ratio between the optical density at D13 and the optical density at D6 larger than 2 was observed for conditions YE, CYE1, CYE2, CYE3, Vitamins 1X, Vitamins 0.5X, Vitamins 0.1X, Biotin, Nicotinic acid, Riboflavin and Thiamine HCl. In contrast, the magnetic response was negative for conditions Vitamins 10X, Vitamins 5X, Calcium pantothenate, Folic acid, Inositol, p-Aminobenzoic acid and Pyridoxine HCl. In conclusion, the yeast extract can be replaced by a single vitamin, which is biotin, riboflavin, nicotinic acid or thiamin HCl. These vitamins yield values of OD_565nmD13_/OD_565nmD6_ of 9.8 (biotin), 4.8 (riboflavin), 2.4 (nicotinic acid), 5.8 (Thiamin HCl) and 95% of magnetic response (table S5). This experiment was performed in triplicate

**Example 3: Determination of the minimal concentrations of the main components of the growth media (sodium lactate, ammonium chloride,** **magnesium sulfate, potassium phosphate), enabling the growth of magnetotactic bacteria and the synthesis of magnetosomes by these bacteria.**

In this example we used non-pharmaceutical grade chemicals for the preparation of the growth media. We varied the concentration of sodium lactate (conditions SL0, SL0.5X, SL0.2X, SL0.1X), ammonium chloride (AC0, AC0.5X, AC0.2X, AC0.1X), magnesium sulfate heptahydrate (MS0, MS0.5X, MS0.2X, MS0.1X), potassium phosphate dibasic (PP0, PP0.5X, PP0.2X, PP0.1X). The chemical compositions and concentrations of the pre-growth and growth media Positive control, SL0, SL0.5X, SL0.2X, SL0.1X, AC0, AC0.5X, AC0.2X, AC0.1X, MS0, MS0.5X, MS0.2X, MS0.1X, PP0, PP0.5X, PP0.2X, PP0.1X, are summarized in Table S6 for 1 liter of growth media. During a first step of the experiment (D1), cryo-stocks of MSR-1 bacteria were grown in 50 mL tubes filled with 8 mL of pre-growth medium and incubated during 6 days until D6 at 29.5°C in an orbital shaker incubator operated at 100 rpm. During a second step, 50 mL tubes were filled with 30 mL of growth medium supplemented with iron citrate (at a concentration of 200µM) acting as the iron source and incubated during 7 days until D13 in the same conditions as in the first step of culture. Samples of 2 mL were taken at the end of each step of culture (D6 and D13) to determine the optical density at 565 nm, OD_565_, at D6 and D13, and the magnetic response of the bacteria, MR, at D13.. For conditions Positive control, PP0.5X, PP0.2X, PP0.1X a growth ratio, or ratio between the optical density measured at D13 and the optical density measured at D6, was larger than 1, and a positive magnetic response was observed at D13. It indicates that these conditions enabled the bacteria to grow and produce magnetosomes. By contrast, for conditions SL0, SL0.5X, SL0.2X, SL0.1X, AC0, AC0.5X, AC0.2X, AC0.1X, MS0, MS0.5X, MS0.2X, MS0.1X, PP0X, the synthesis of the magnetosomes was very low (magnetic response < 50 %). In conclusion, the concentration of potassium phosphate can be reduced in the growth medium by a factor of 2 or 5 without affecting the growth and production of magnetosomes. Indeed, these conditions yield values of OD_565nmD13_/OD_565nmD6_ of 1.5 (condition PP0.5X), 2.1 (conditions PP0.2X and PP0.1X), and a positive magnetic response among bacteria . By contrast, the concentrations of the other chemicals of the medium (ammonium chloride, sodium lactate, magnesium phosphate) could not be reduced without significantly affecting the growth and/or the magnetic response of MSR-1 magnetotactic bacteria (Table S7). This experiment was performed in triplicate

**Example 4: Determination of the minimal concentrations of the main components of the feeding medium (Lactate, ammonia, magnesium sulfate, potassium phosphate, iron chloride), enabling the growth of magnetotactic bacteria and the synthesis of magnetosomes by these bacteria.**

We varied the concentration of lactate (LA0.5X, LA0.2X, LA0.1X), ammonia (A0.5X, A0.2X, A0.1X), magnesium sulfate heptahydrate (MS0.5X, MS0.2X, MS0.1X), potassium phosphate dibasic (PP0.2X, PP0.1X), iron chloride (IC0.5X, IC0.2X, IC0.1X). The chemical compositions and concentrations of the Feeding media (Zhang et al. 2011) FBZ, LA0.5X, LA0.2X, LA0.1X, A0.5X, A0.2X, A0.1X, MS0.5X, MS0.2X, MS0.1X, PP0.2X, PP0.1X, IC0.5X, IC0.2X, IC0.1X are summarized in table S8 for 1 liter of growth media. All chemicals were pharmaceutical grade (Merck). During a first step of the experiment (D1), cryo-stocks of MSR-1 bacteria were grown in 500 mL bottles filled with 250mL of minimal pre-growth medium and incubated during 6 days until D6 at 29.5 °C in an orbital shaker incubator operating at 100 rpm. During a second step, cells were transferred in DASGIP Parallel 1.5 L Bioreactor System filled with 800 mL of minimal growth medium. Fermentation was carried out at 29.5 °C initial OD_565_ ~ 0.09 under agitation at 200 rpm during 2 days until D8 at pH maintained at 6.9 by adding an acidic fed-batch medium (Table S8) containing FeCl_3_.6H_2_O as the iron source. Growth of MSR-1 bacteria was stimulated by bubbling oxygen in the growth medium at a percentage kept below 1 % to enable magnetosome synthesis. Temperature, agitation speed, pH, feeding pump flow and oxygen concentration were monitored and adjusted using a DASGIP controller and a DASware software from Eppendorf. Samples of 2mL were taken at D6, D7 and D8. For conditions FBZ, MS0.5X, MS0.2X, MS0.1X, PP0.2X, PP0.1X the OD_565_ measured at D7, *i.e.* 24H after the beginning of the growth step, was ~0.5 and a positive magnetic response was observed. It indicates that these conditions enabled the bacteria to grow and produce magnetosomes. By contrast, for conditions LA0.5X, LA0.2X, LA0.1X, A0.5X, A0.2X, A0.1X, IC0.5X, IC0.2X, IC0.1X the OD_565_ was larger than 0.3 and a negative magnetic response was observed. It indicates that these conditions enabled the bacteria to grow but impair the production of magnetosomes. At D8, *i.e.* 48H after the beginning of the growth step, for conditions IC0.5X, IC0.2X, IC0.1X the OD_565_ was larger than 1 and a negative magnetic response was observed. For conditions L0.5X, L0.2X, L0.1X the OD_565_ was lower than 1 and a positive magnetic response was observed. By contrast for conditions FBZ, A0.5X, A0.2X, A0.1X, MS0.5X, MS0.2X, MS0.1X, PP0.2X, PP0.1X the OD_565_ was larger than 1 and a positive response was observed. In conclusion, the concentration of magnesium sulfate and potassium phosphate can be reduced in the feeding medium by a factor of 2 or 5 without affecting the growth and production of magnetosomes. By contrast the concentrations of the other chemicals of the feeding medium (lactate, ammonia, iron chloride) could not be reduced without affecting the growth and/or the magnetic response of MSR-1 magnetotactic bacteria. This experiment was performed in triplicate

**SUPPLEMENTARY TABLES:**

**Table S1**: Chemical compositions in one liter of growth medium of the different mineral elixirs (ME1, ME2, ME3, ME4, ME5 and ME6) tested.

**Table S2**: For example 1, optical density measured at the end of the pre-growth step, 6 days following the beginning of growth, OD_D6_, or 13 days following the beginning of growth, OD_D13_, ratio OD_D13_/OD_D6_, and positive (+) or negative (-) magnetic response, for the different mineral elixir tested.

**Table S3**: Composition in one liter of growth medium of yeast extract and chemical equivalents of yeast extract (YE, CYE1, CYE2, CYE3) tested.

**Table S4**: Compositions in one liter of growth medium of the different vitamin cocktails and individual vitamins (Vitamins1X, Vitamins 5X, Vitamins 10X, Vitamins 5X, Vitamins 0.5X, Vitamins 0.1X, Biotin, Calcium pantothenate, Folic acid, Inositol, Nicotinic acid, p-Aminobenzoic acid, Pyridoxine HCl, Riboflavin, Thiamine HCl) tested.

**Table S5**: For example 2, optical density measured at the end of the pre-growth step, 6 days following the beginning of growth, OD_D6_, or 13 days following the beginning of growth, OD_D13_, ratio OD_D13_/OD_D6_, and positive (+) or negative (-) magnetic response, for the different mineral yeast extracts, vitamin coktails and individual vitamins tested.

**Table S6**: Compositions in one liter of deionized water of the pre-growth and growth media used to grow MSR-1 magnetotactic bacteria in 50 milliliter tubes following example 3. In this example, 4 different concentrations of sodium lactate (SL0, SL0.5X, SL0.2X, SL0.1X), ammonium chloride (AC0, AC0.5X, AC0.2X, AC0.1X), Magnesium sulfate heptahydrate (MS0, MS0.5X, MS0.2X, MS0.1X), Potassium phosphate dibasic (PP0, PP0.5X, PP0.2X, PP0.1X) were tested.

**Table S7**: For example 3, optical density measured at the end of the pre-growth step, 6 days following the beginning of growth, OD_D6_, or 13 days following the beginning of growth, OD_D13_, ratio OD_D13_/OD_D6_, and positive (+) or negative (-) magnetic response, for 4 different concentrations of sodium lactate (SL0, SL0.5X, SL0.2X, SL0.1X), ammonium chloride (AC0, AC0.5X, AC0.2X, AC0.1X), magnesium sulfate heptahydrate (MS0, MS0.5X, MS0.2X, MSO.1X), Potassium phosphate dibasic (PP0, PP0.5X, PP0.2X, PP0.1X) tested.

**Table S8**: Compositions in one liter of deionized water of the feeding media used to grow MSR-1 magnetotactic bacteria in 1.5L fermenter following example 4. In this example, 3 different concentrations of lactic acid (LA0.5X, LA0.2X, LA0.1X), ammonia (AC0, AC0.5X, AC0.2X, AC0.1X), Magnesium sulfate heptahydrate (MS0.5X, MS0.2X, MS0.1X), iron chloride hexahydrate (IC0.5X, IC0.2X, IC0.1X) and 2 concentrations of Potassium phosphate dibasic (PP0.2X, PP0.1X) were tested.

**Table S9**: For example 4, optical density measured at the end of the pre-growth step, 6 days following the beginning of growth, OD_D6_, or 7 days following the beginning of growth, OD_D7_, or 8 days following the beginning of growth, OD_D8_, and positive (+) or negative (-) magnetic response for 3 different concentrations of lactic acid (LA0.5X, LA0.2X, LA0.1X), ammonia (AC0, AC0.5X, AC0.2X, AC0.1X), Magnesium sulfate heptahydrate (MS0.5X, MS0.2X, MS0.1X), iron chloride hexahydrate (IC0.5X, IC0.2X, IC0.1X) and 2 concentrations of Potassium phosphate dibasic (PP0.2X, PP0.1X) tested.

**SUPPLEMENTARY FIGURE:**

**Figure S1**: Boxplot, obtained from TEM images, of magnetosomes size (n=325) distribution (A) and number of magnetosomes (n=200) per cell (B) at 0, 1, 3, 10, 23, 26, 44, 47 and 50H of culture by MSR-1 magnetotactic bacteria in 70L fermenter. The typical construction of the boxplot displays the full range of variation (from 5th to 95th percentiles), the likely range of variation (the 1st and the 3rd quartiles), the median (black dash) and mean (black square) values. Statistical significance of magnetosomes size distribution (A) and the number of magnetosomes per cell (B) were evaluated using T tests with Origin 6.1 software. Parameters are expressed as p-values (p<0.001) and compared pair-wise at 3, 26 and 50H of culture.


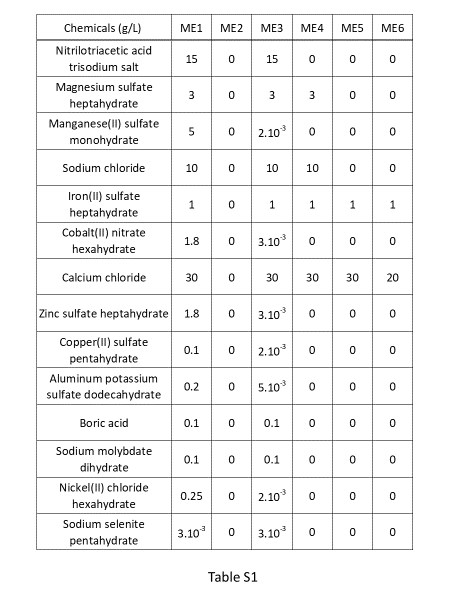


Table S1

Table S1


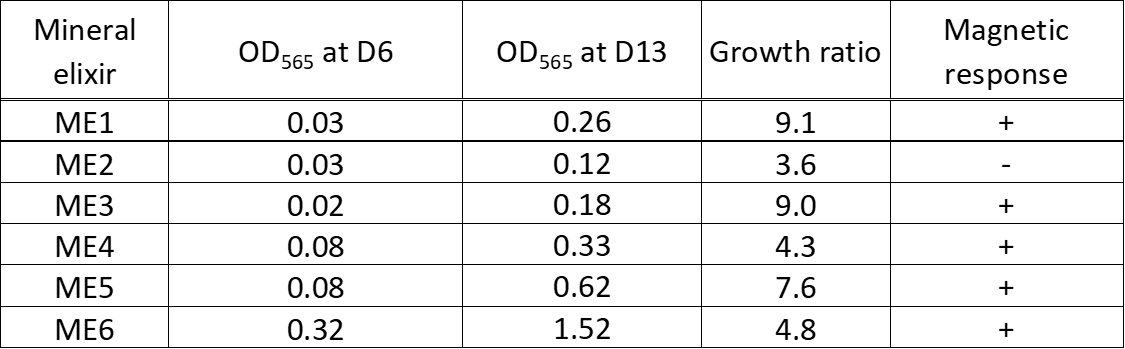


Table S2


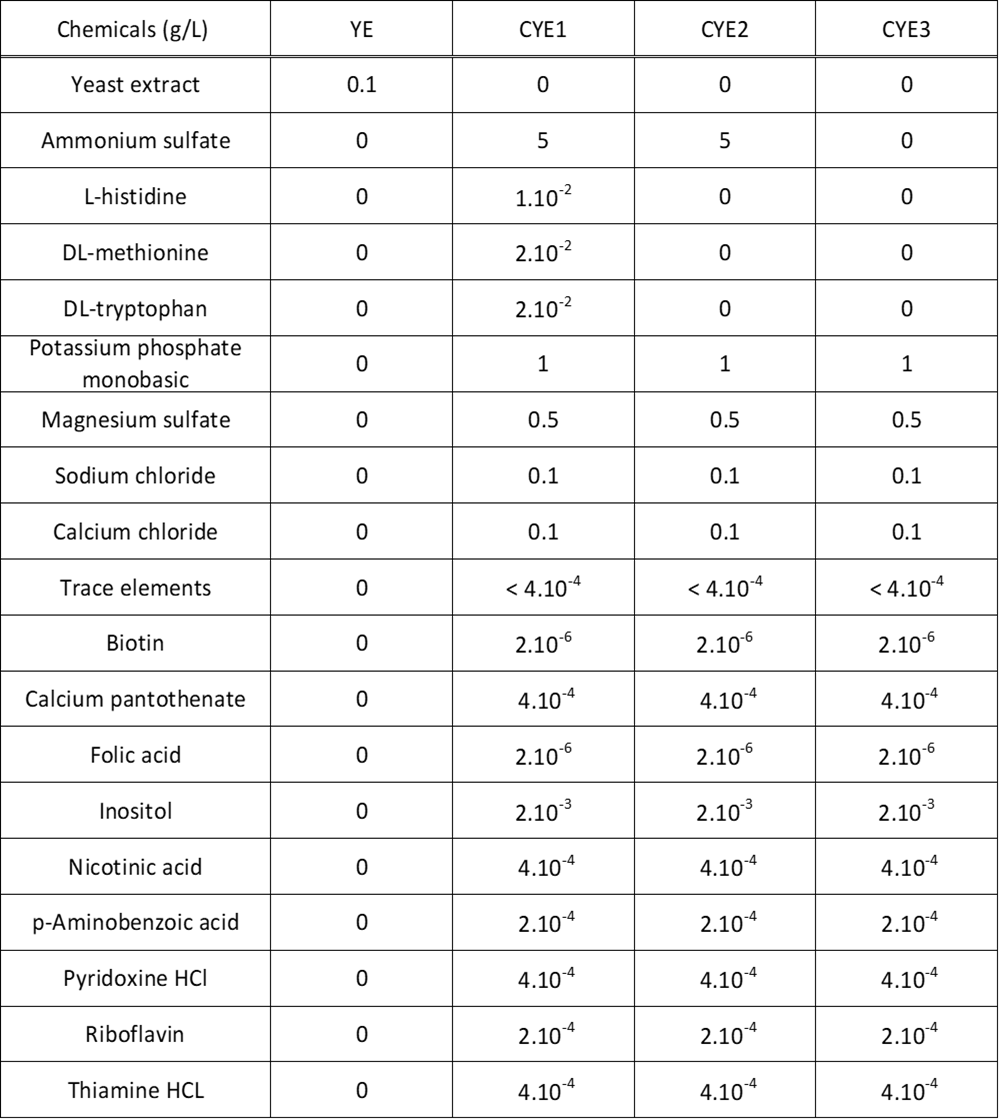


Table S3

Table S3


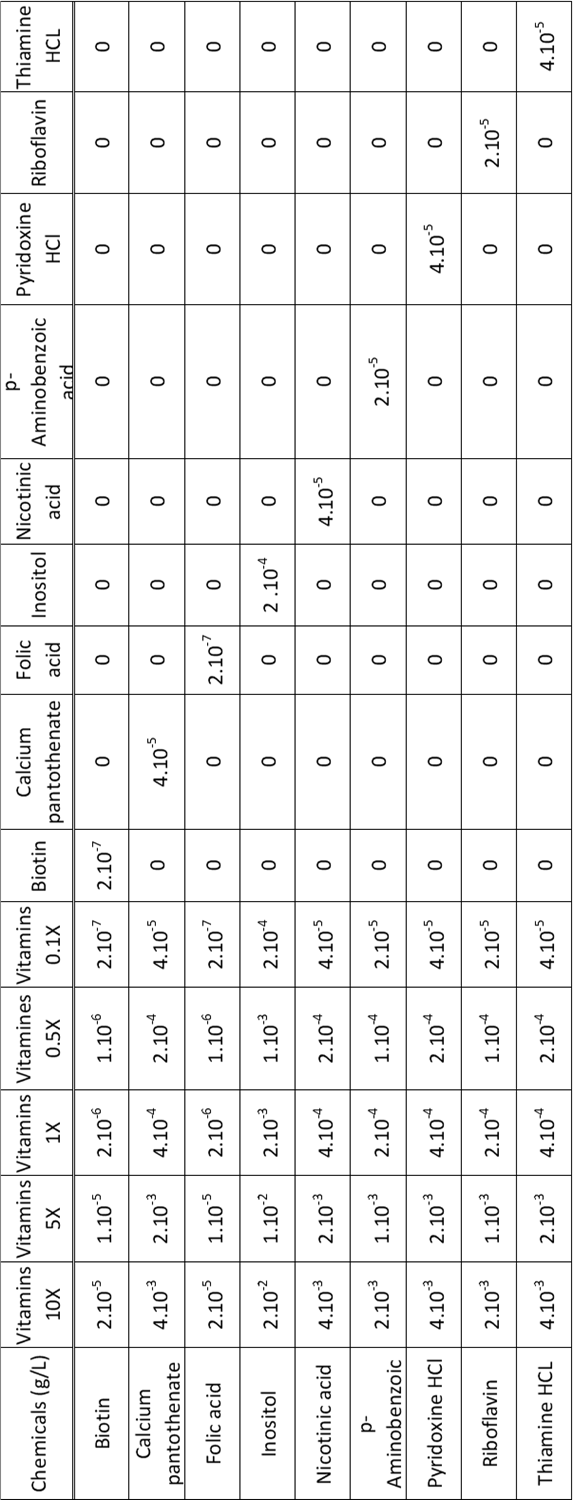


Table S4


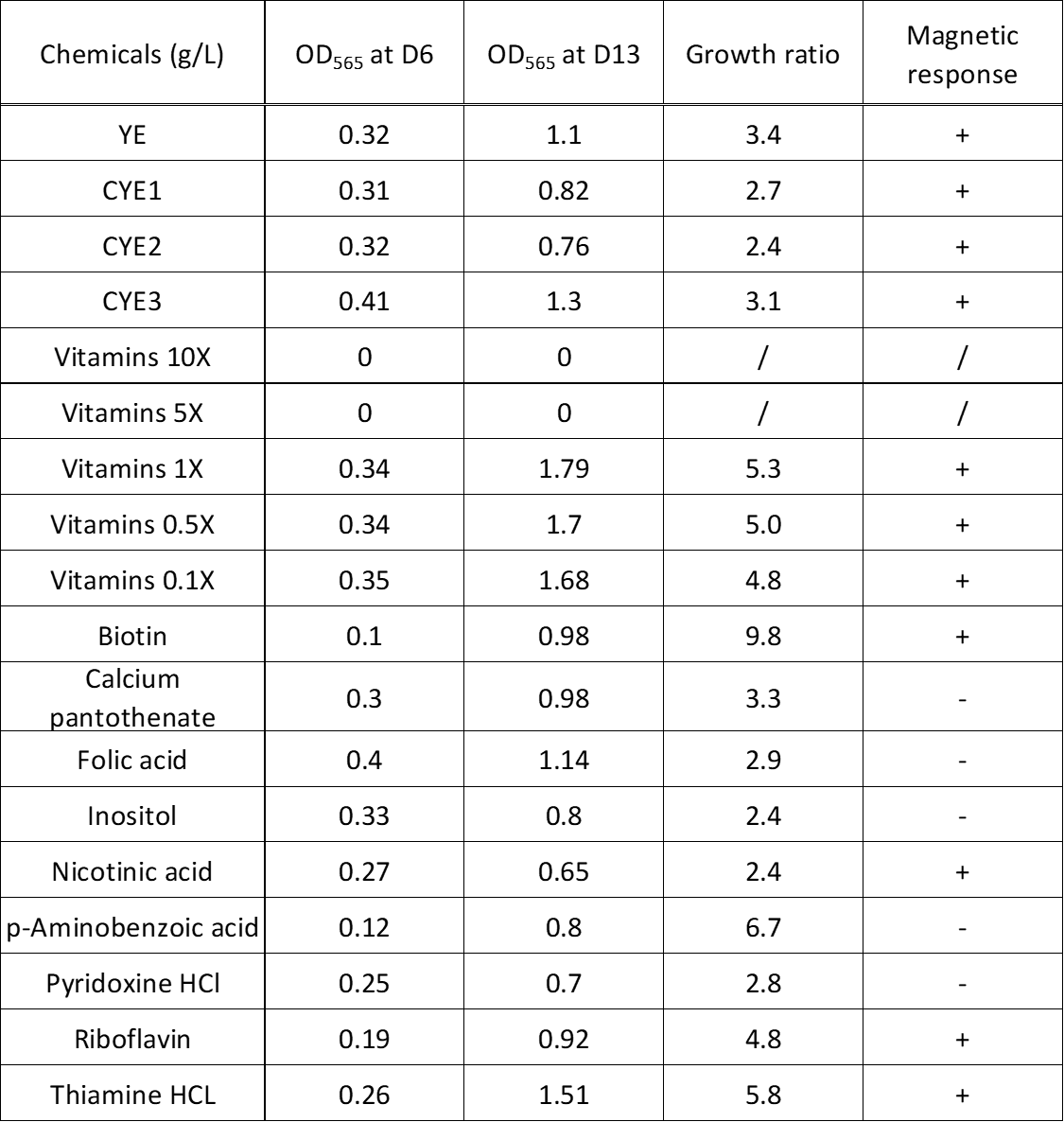


Table S5


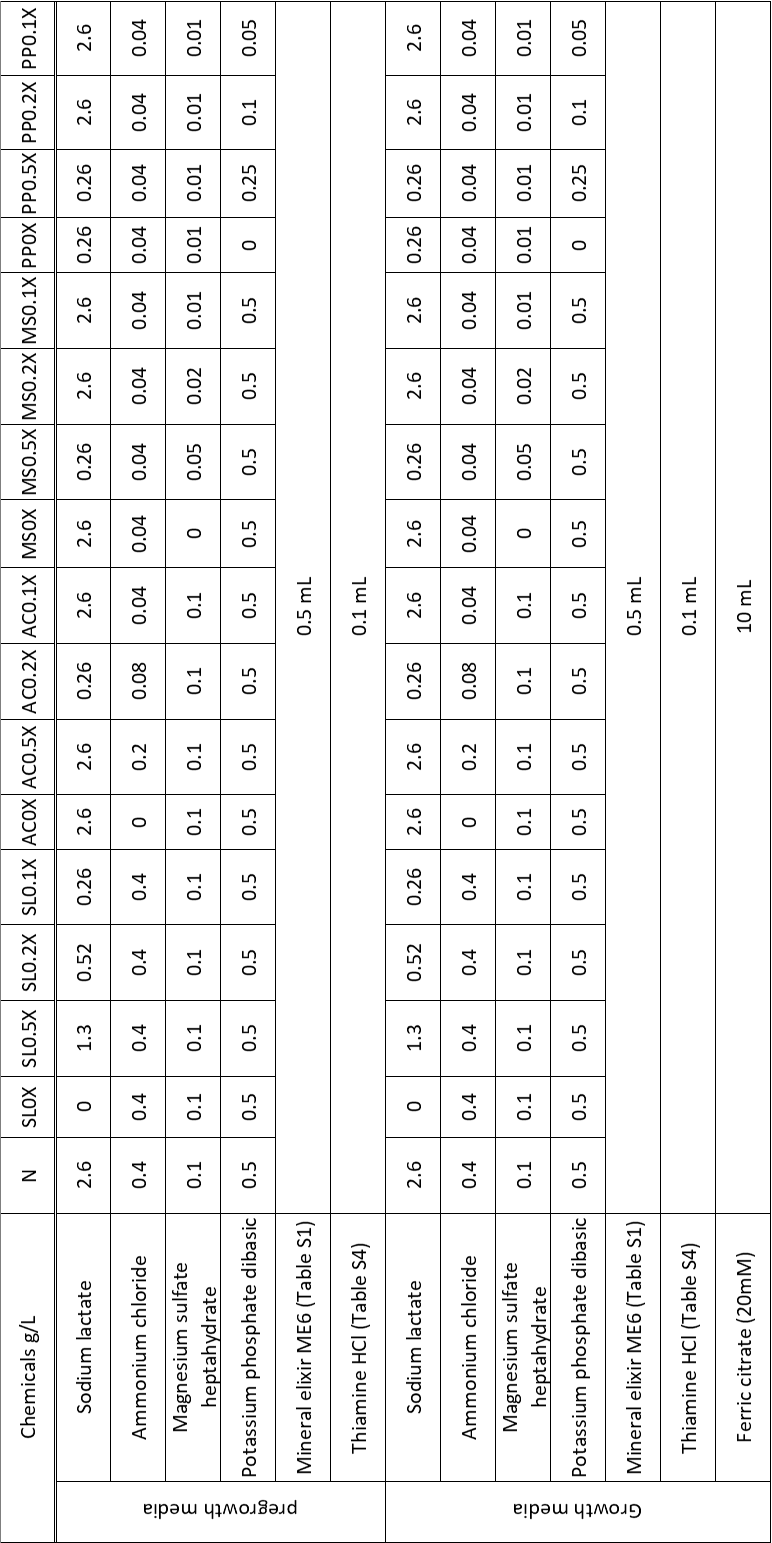


Table S6


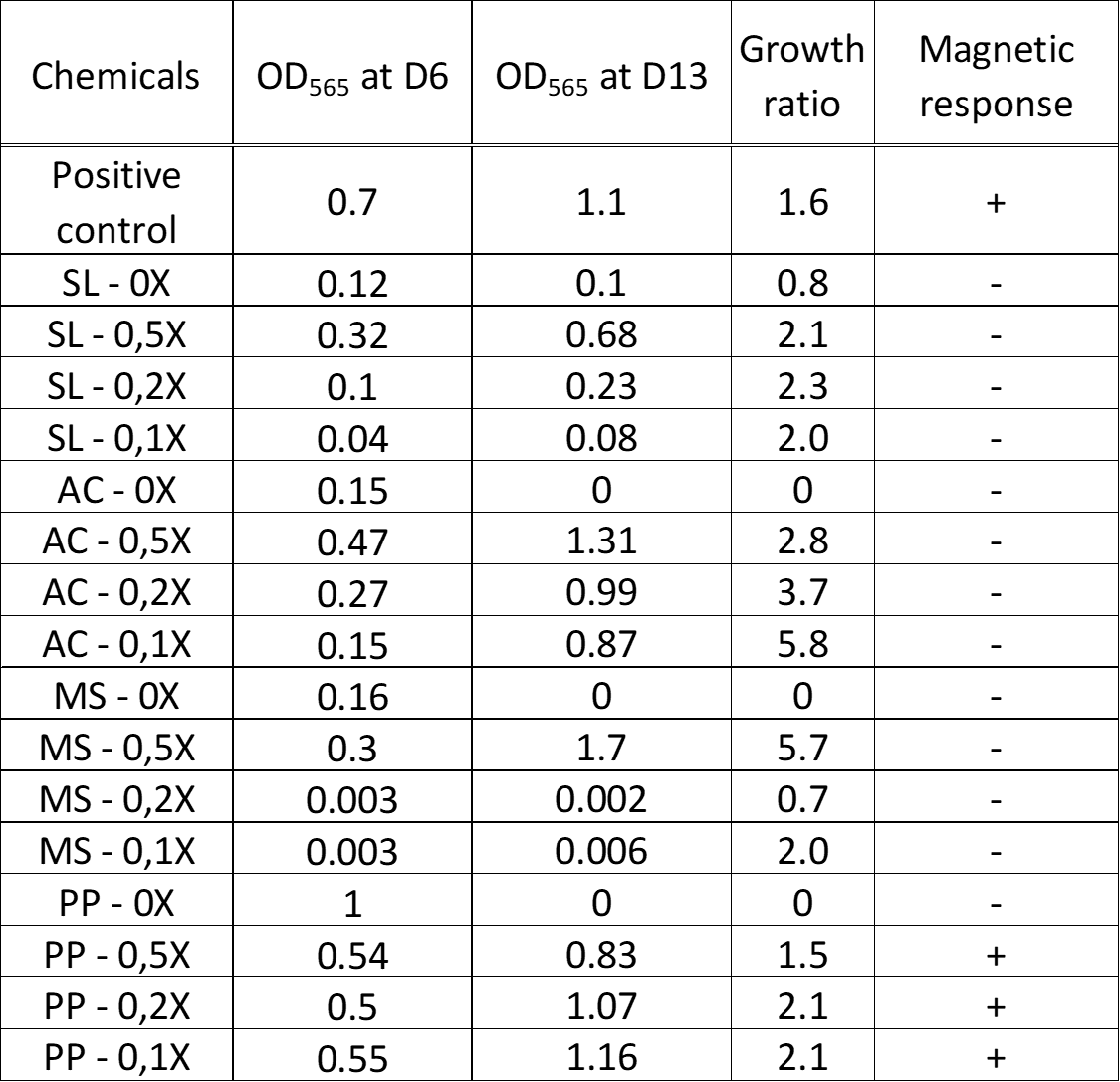


Table S7

Table S7


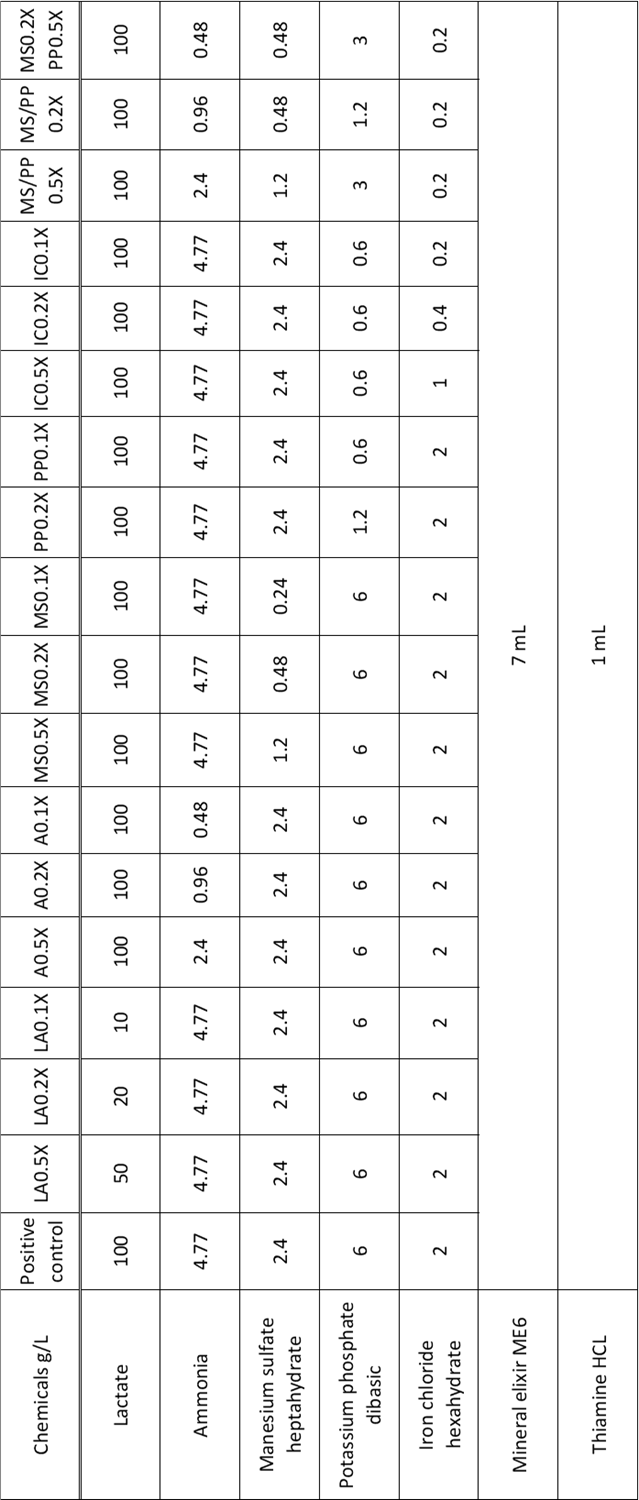


Table S8


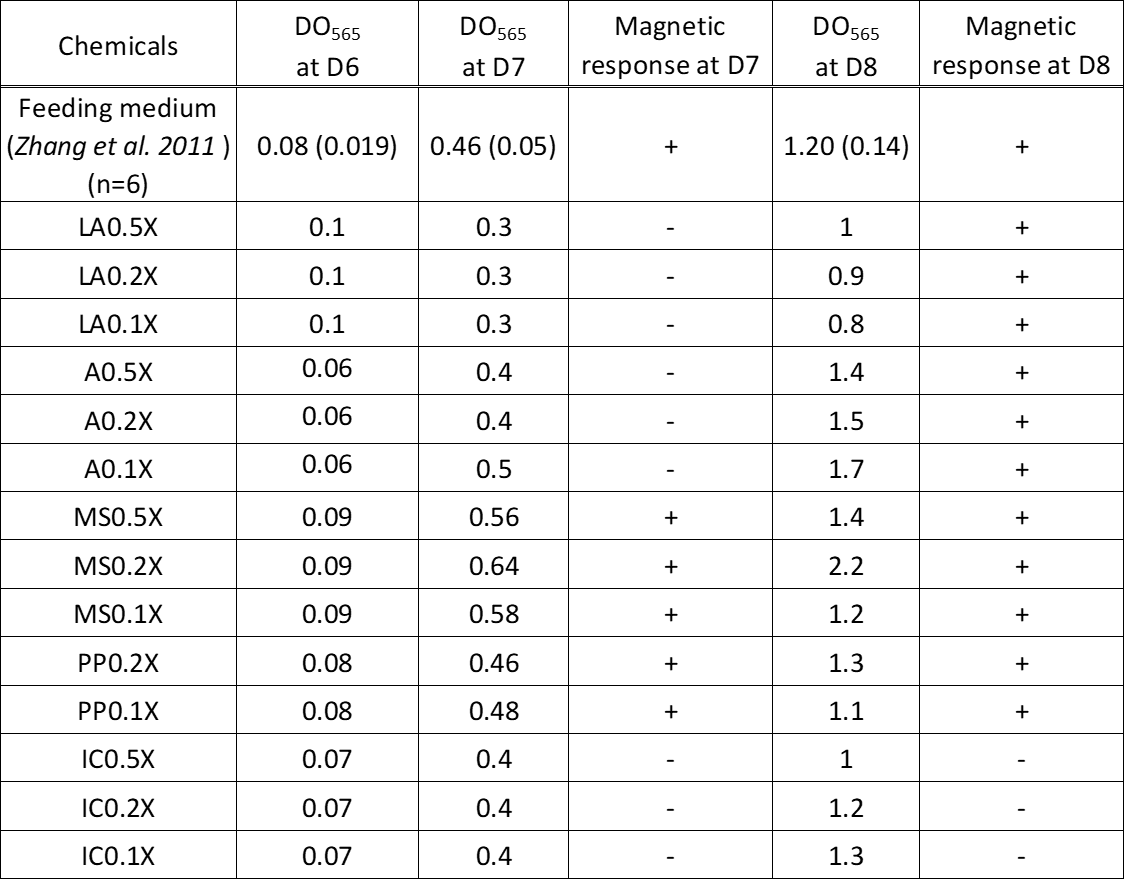


Table S9


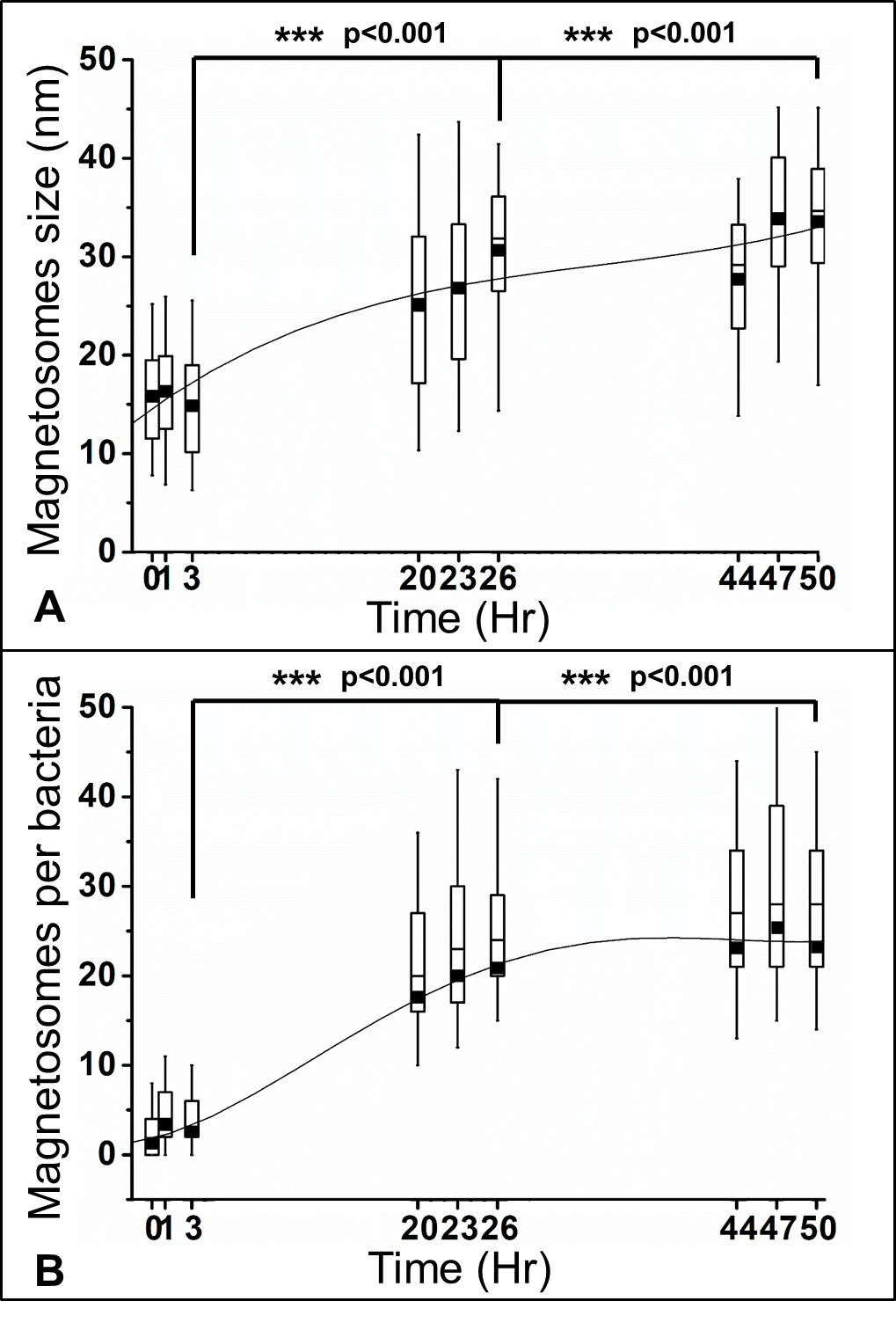


Figure S1

Figure S1

Figure S1
